# Evaluation of Alysis-001 cuffless blood pressure estimation algorithm against the European Society of Hypertension awake/asleep test criteria in hypertensive patients

**DOI:** 10.1038/s41440-026-02728-z

**Published:** 2026-08-21

**Authors:** Kazuhiro Hongyo, Atsushi Hirayama, Kosuke Shimizu, Taisuke Ueno, Taku Fujimoto, Akitoshi Hara, Hiroshi Koriyama, Yoichi Takami, Yoshio Iwashima, Ryo Kawasaki, Koichi Yamamoto

**Affiliations:** 1 Department of Geriatric and General Medicine, Graduate School of Medicine, The University of Osaka, Suita, Japan; 2 Division of Public Health, Department of Social Medicine, Graduate School of Medicine, The University of Osaka, Suita, Japan; 3 Arblet Inc., Tokyo, Japan

**Keywords:** Ambulatory blood pressure monitoring, Implemental hypertension, Cuffless blood pressure algorithm, Digital hypertension

## Abstract

Accurate ambulatory blood pressure monitoring (ABPM) is essential for cardiovascular risk assessment and hypertension management, but conventional cuff-based ABPM causes discomfort and sleep disturbance, potentially affecting measurement reliability. The European Society of Hypertension (ESH) established evaluation criteria for cuffless blood pressure devices, with the awake/asleep test as the primary evaluation test for automated wearable devices. This study evaluated whether Alysis-001, a software-based cuffless blood pressure estimation algorithm using sensor data from a wrist-worn device, meets ESH awake/asleep test criteria in hypertensive patients under two data extraction strategies: (1) arm-stabilized periods only ( ≥ 10 s static); (2) including low-intensity activity (high-intensity excluded). We prospectively enrolled 92 hypertensive outpatients receiving antihypertensive treatment who simultaneously wore Alysis Wear and underwent conventional 24-h ABPM; 78 completed the study. Under both strategies, Alysis-001 demonstrated excellent agreement with reference ABPM across all measurement periods, with intraclass correlation coefficients exceeding 0.70 for systolic and diastolic blood pressure. Alysis-001 successfully met all ESH awake/asleep test criteria under both strategies: mean difference between awake-asleep blood pressure changes ≤5 mmHg, standard deviation ≤8 mmHg, and correlation coefficients ≥0.70 for both systolic and diastolic blood pressure. Alysis-001 also demonstrated high sensitivity, specificity, and accuracy in identifying non-dippers under both strategies. To our knowledge, this represents the first evaluation of a Device Type 3 cuffless blood pressure algorithm (automated, wearable, demographic-calibrated, not at heart level) meeting ESH awake/asleep test criteria, suggesting Alysis-001’s potential as a clinically viable alternative for ambulatory blood pressure monitoring in hypertensive patients.

**Figure:**
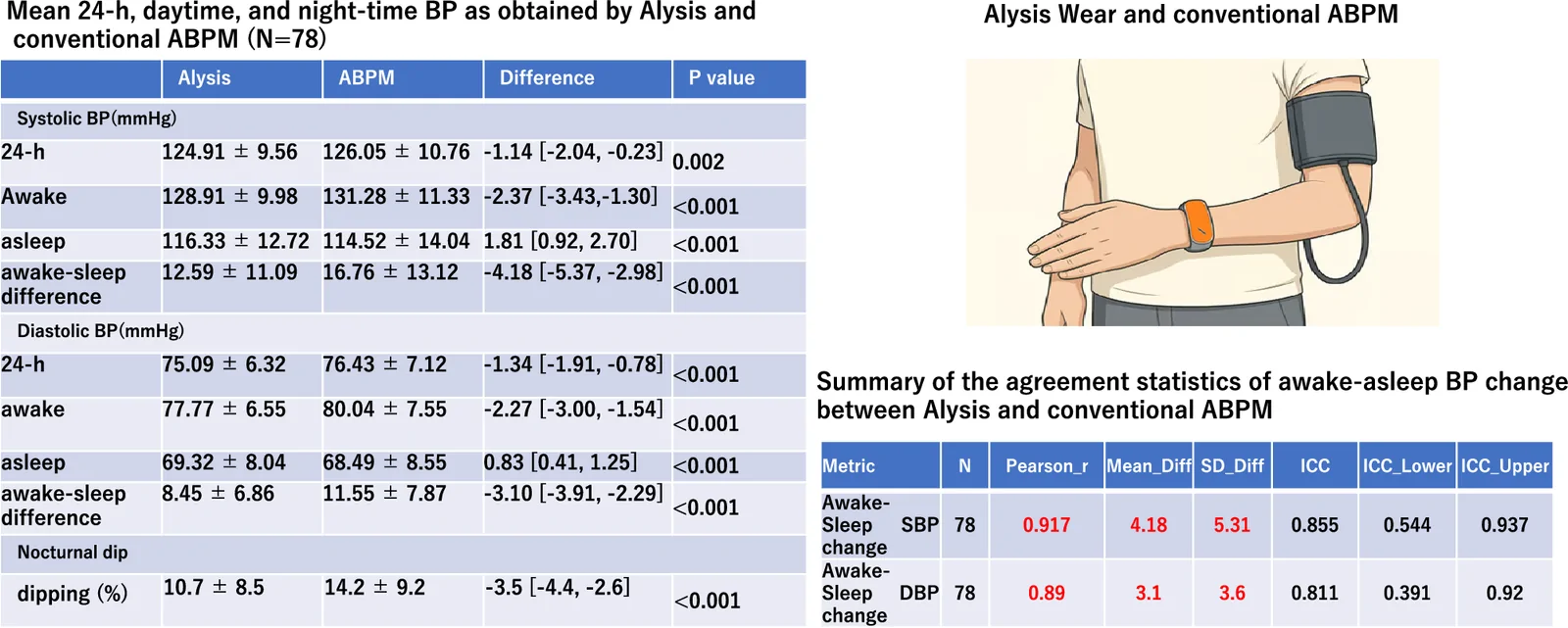

## Introduction

Hypertension is a major risk factor for cardiovascular diseases, including myocardial infarction, heart failure, and stroke [1, 2]. Blood pressure (BP) fluctuates throughout the day depending on environmental factors such as rest or physical exertion. As clinic-based measurements may not accurately reflect true blood pressure status, ambulatory BP monitoring (ABPM) is preferred for evaluating cardiovascular risks [3–5].

24-h ABPM is essential for detecting white-coat and masked hypertension, evaluating nocturnal hypertension and morning surge, and enabling accurate cardiovascular risk stratification [5]. However, conventional cuff-based ABPM causes discomfort due to intermittent arm cuff inflation, potentially compromising measurement reliability during sleep by triggering awakening responses that elevate BP. Therefore, cuffless ABPM methods that reduce patient stress are highly needed.

Cuffless methods enable continuous BP monitoring and can capture subtle fluctuations associated with daily activities, advancing hypertension management. However, as these systems estimate BP from sensor data such as pulse waves, rigorous evaluation is essential to ensure clinical reliability comparable to traditional cuff-based monitors [6].

The European Society of Hypertension (ESH) Working Group established comprehensive evaluation procedures specifically for cuffless BP devices [7]. These recommendations define six tests: static test, device position test, treatment test, awake/asleep test, exercise test, and recalibration test. For automated wearable devices requiring individual-user calibration, the awake/asleep test is designated as the “primary test” to be performed first [7]. This test assesses the system’s ability to track clinically important BP changes during sleep by comparing with the reference 24-h ABPM. It is considered the most clinically meaningful evaluation because it evaluates diurnal BP tracking while indirectly assessing accuracy under varying positions, activities, and motion during daily life [7]. ESH recommends that additional evaluation tests be performed only after successfully passing this primary test.

An important consideration for cuffless wearable devices is the definition of valid measurement periods. While conventional cuff-based ABPM measures BP at predetermined intervals regardless of activity state, cuffless devices continuously collect sensor data and must determine when to estimate BP. To ensure measurement reliability, many systems restrict BP estimation to periods when motion artifacts are minimal. However, defining optimal data extraction strategies—balancing measurement accuracy with real-world applicability—remains an important evaluation question. The awake/asleep test provides an ideal framework to compare different data extraction approaches, as it evaluates performance across the full spectrum of daily activities captured during 24-h monitoring.

In this study, we evaluated Alysis-001, a BP estimation software algorithm (Software as a Medical Device) developed by Arblet Inc. The algorithm was trained on independent datasets comprising 953 seated-rest and 150 treadmill-walking recordings with reference BP measured by a commercially available aneroid sphygmomanometer and ABPM, respectively. We assessed whether Alysis-001 demonstrates accuracy comparable to conventional cuff-based ABPM in tracking awake-to-asleep BP changes according to ESH awake/asleep test criteria in patients with hypertension under two data extraction strategies: (1) arm-stabilized periods only ( ≥ 10 s static, detected by inertial sensors); and (2) all available measurements, including resting and low-intensity activity periods (high-intensity activity excluded). This comparison allows evaluation of how measurement selection criteria affect clinical performance in tracking diurnal BP variation.

## Methods

### Study design

This prospective observational study was conducted at the University of Osaka Hospital in Osaka, Japan, between January and April 2025. The study protocol was approved by the Institutional Review Board of Osaka University Hospital (approval number: 24028(T1)-5; approval date: 27/11/2024) and was conducted in accordance with the Declaration of Helsinki. All participants provided written informed consent prior to enrollment.

### Device description

#### Structure and measurement principle

Alysis Wear is a wrist-worn wearable device developed by Arblet Inc. (Tokyo, Japan). It incorporates multiple sensors that provide the data used to estimate BP noninvasively, including:

- A photoplethysmography (PPG) sensor that emits green, red, and infrared light to detect changes in blood volume through reflected light.
- An electro-potential sensor that measures bioelectrical signals caused by pulsatile blood volume changes near surface arteries.
- Inertial sensors (accelerometers and gyroscopes) to detect posture and motion.
- A temperature sensor that monitors skin temperature to account for thermal influences on blood flow.

The Alysis-001 algorithm estimates BP based on biomechanical principles using the Moens–Korteweg equation, which expresses the relationship between pulse wave velocity v and arterial wall elasticity:

$$v=\sqrt{\frac{{Eh}}{2r\rho }}$$

where r is the arterial radius, h is the arterial wall thickness, E is the incremental elastic modulus of the artery, and ρ is the blood density. Since the elastic E modulus is positively correlated with BP P, it can be expressed as:

$$E={E}_{0}{e}^{\alpha P}$$

where ${E}_{0}$ is the initial elastic modulus, and α is a constant derived empirically. Combining these equations, the algorithm estimates BP using three primary physiological factors: (1) Blood flow velocity derived from the time difference between the electrical and PPG signals; (2) Arterial volume estimated from pulse waveform morphology; (3) Arterial elasticity calculated via second-order derivatives of the PPG signal. This model allows Alysis-001 to infer BP from pulse wave characteristics without the need for cuff-based occlusion.

The Alysis-001 algorithm utilizes demographic parameters—specifically age, height, and body weight—as auxiliary inputs to refine BP estimation. These variables are incorporated to account for inter-individual differences in vascular characteristics, such as arterial stiffness and pulse wave velocity. However, BP values are not determined solely by these demographic factors; they are integrated into a multi-parametric model dominated by signal-derived features. The alfortihmic framework is designed to optimally weight the extracted physiological features against the demographic auxiliary inputs to calculate BP values. The algorithm does not rely on individual calibrations using cuff-based measurements. The baseline characteristics of the independent datasets used to train this algorithm are summarized in Supplementary Table 1.

### Motion and posture compensation

To improve measurement reliability, inertial data are used to classify activity levels into resting, low-intensity, and high-intensity categories. BP estimation is suspended during high-intensity activity. Furthermore, the inclination angle and user height are used to correct for the vertical difference between the wrist and heart, minimizing hydrostatic pressure error.

### Activity level classification

Alysis Wear continuously monitors physical activity using built-in 3-axis accelerometers and gyroscopes. Activity levels are classified into three categories based on inertial sensor data:

- Resting: Periods of sustained stillness ( ≥ 10 s) with minimal motion artifacts, typically corresponding to sitting, lying down, or standing still.
- Low-intensity activity: Periods of light movement such as slow walking, arm gestures during conversation, or other non-strenuous activities that do not generate significant motion artifacts.
- High-intensity activity: Periods of vigorous movement such as fast walking, running, climbing stairs, or exercise that generate substantial motion artifacts and compromise signal quality.

BP estimation is performed during resting and low-intensity activity periods but is automatically suspended during high-intensity activity to maintain measurement reliability. This classification enables two data extraction strategies:

- Strategy 1 (Arm-stabilized only): BP estimates obtained only during resting periods ( ≥ 10 s static).
- Strategy 2 (Including low-intensity activity): BP estimates obtained during both resting and low-intensity activity periods (high-intensity activity excluded).

### Alysis-001 algorithm development

Prior to this evaluation, the Alysis-001 algorithm was developed using independent datasets collected in separate cohorts with no overlap with the participants in the present study. The training datasets comprised 953 seated-rest recordings, obtained under the same resting protocol as in the present trial, and 150 treadmill-walking recordings acquired at a comfortable, non-exertional pace. For the walking cohort, participants walked continuously on a treadmill without becoming breathless, and physiological signals were collected throughout the session. Reference BP values for all training data were measured 7 times per participant by a commercially available aneroid sphygmomanometer and ambulatory BP monitor (ABPM), respectively. These datasets were used exclusively for model development and training of the Alysis-001 algorithm.

### Participants

This study enrolled 92 hypertensive outpatients aged 18 years or older who were receiving antihypertensive treatment. All participants had a diagnosis of hypertension and were on stable medication regimens. Exclusion criteria included: (1) presence of chronic arrhythmia; (2) wrist skin conditions such as wounds, burns, surface irregularities, or very high melanin levels (e.g., Fitzpatrick type V-VI) where optical sensor signals could not be reliably acquired; (3) limb amputation or presence of a vascular shunt in the left arm; (4) inability to wear the study device properly (wrist circumference <130 mm or >195 mm); and (5) incompatibility with ambulatory BP monitoring (ABPM) cuff size (upper arm circumference <200 mm or >420 mm).

Demographic data collected included age, sex, height, weight, body mass index (BMI), antihypertensive medication use, and hypertension status. All participants provided written informed consent prior to participation.

### Equipment

#### Reference ABPM device

Twenty-four-hour reference BP measurements were obtained using a validated oscillometric ABPM device TM-2441 in accordance with European Society of Hypertension (ESH) guidelines. The device was programmed to measure BP at 30-minute intervals throughout the 25-h monitoring period. To minimize the potential influence of the initial alerting reaction, the first hour of recordings was discarded, and BP values from the following continuous 24-h period were analyzed. Awake and asleep periods were defined based on participant-reported in-bed and out-of-bed times recorded in a diary. A valid 24-h ABPM session requires at least 25 valid awake readings and 12 valid asleep readings, with at least one valid reading every 2 h.

#### Cuffless BP monitoring system

**Fig. 1:**
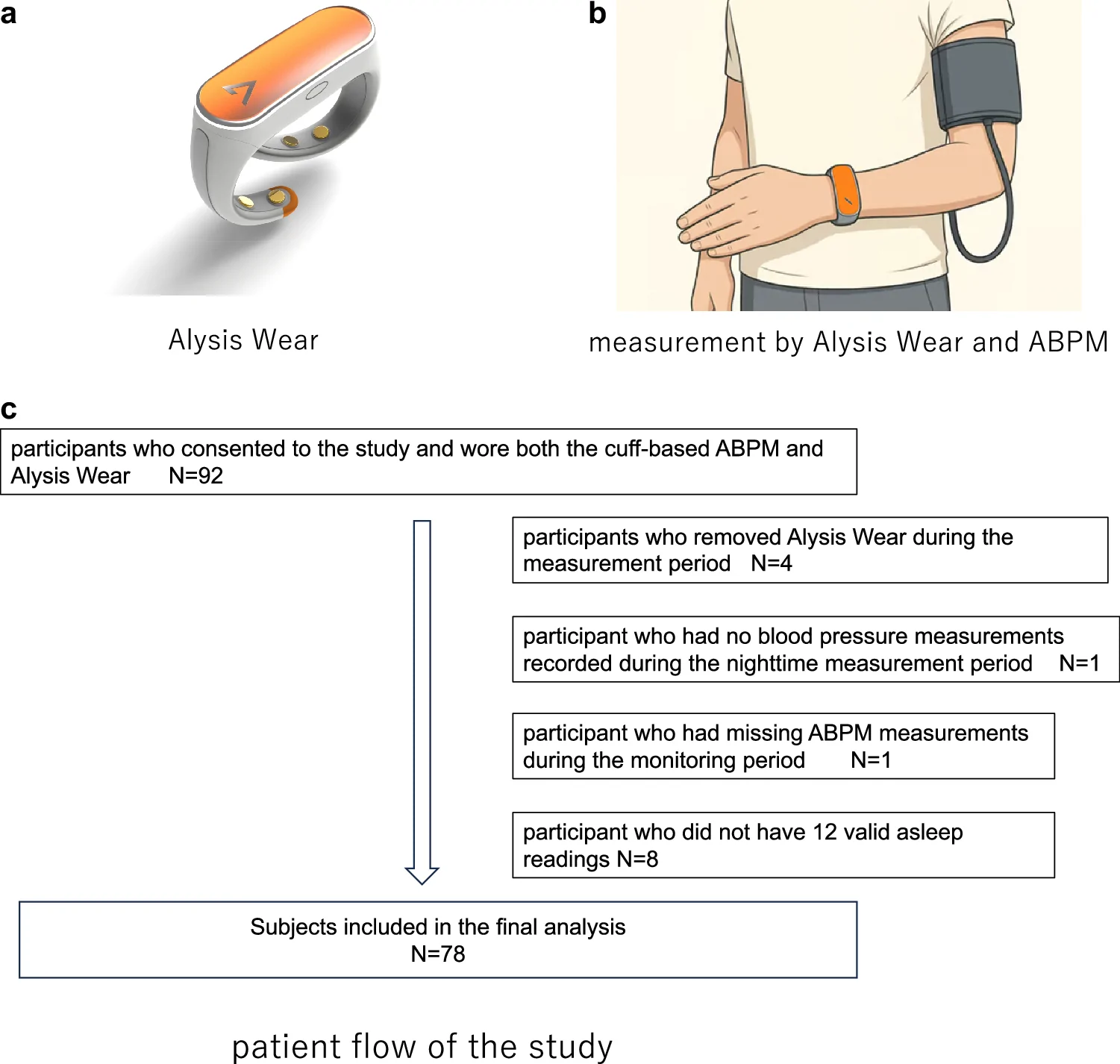
**a** Alysis Wear. A picture of the Alysis Wear is shown. **b** Measurement by Analysis Wear and ABPM. Participants wore both the reference ABPM device on the upper arm and the Alysis Wear device on the ipsilateral wrist simultaneously for a continuous 24-h period. **c** Patient flow of the study. 92 hypertensive patients consented and wore both devices, and 78 were included in the final analysis after exclusion of four participants who removed Alysis Wear during monitoring, one participant without nighttime measurements, one participant with missing ABPM data and eight participants who did not have 12 valid asleep readings

Cuffless BP measurements were obtained using Alysis Wear (Arblet Inc., Tokyo, Japan) in conjunction with the Alysis-001 algorithm, a Software as a Medical Device (SaMD) approved by the Japanese Pharmaceuticals and Medical Devices Agency (Approval no. 30600BZX00212000) (Fig. 1a). The device was securely fastened to the participant’s wrist on the ipsilateral arm relative to the ABPM cuff, allowing simultaneous measurements (Fig. 1b). Prior to each measurement session, the device’s battery level was verified to prevent technical errors.

#### Measurement procedure

Participants were instructed to wear both the reference ABPM device on the upper arm and the Alysis Wear device on the ipsilateral wrist simultaneously for a continuous 24-h period. Participants were asked to maintain their normal daily activities during the monitoring period and to record their in-bed and out-of-bed times in a diary to define awake and asleep periods.

The reference ABPM device was programmed to inflate every 30 minutes throughout the 24-h period. Alysis Wear operated on an intermittent 60-s cycle, acquiring physiological sensor data (PPG, electro-potential, inertial, and temperature signals) at a sampling rate of 51.2 Hz for a fixed 10-second duration, and suspending sensor measurement for the remaining 50 s of each minute. BP estimates from Alysis-001 were generated using two data extraction strategies:

- Strategy 1 (Arm-stabilized only): BP estimates were generated only if the wrist remained stable with minimal motion artifacts during the 10-second measurement window, as detected by the inertial sensors.
- Strategy 2 (Including low-intensity activity): BP estimates were generated during both resting periods and low-intensity activity periods. High-intensity activity periods were automatically excluded to maintain measurement reliability and minimize motion artifacts.

For the cuffless wearable device, BP estimates derived from the above strategies were further aggregated into 30-minute intervals. To ensure comparability with ABPM, which provides intermittent measurements at predefined intervals, each 30-minute aggregated value was treated as a single BP reading for the calculation of awake, sleep, and 24-h averages. Only BP estimates that satisfied the device’s internal signal quality criteria were included in the analysis. This approach aligns the temporal resolution of the wearable-derived BP data with that of ABPM measurements.

### Data analysis

#### BP measurements

For the Alysis Wear device, the continuous signals were segmented beat-by-beat within each 10-s measurement window. After an internal signal quality assessment, valid beat-by-beat values were averaged to generate 1-minute BP estimates. To ensure comparability with the reference ABPM device, these estimates were subsequently aggregated into 30-minute intervals, with each aggregated value treated as a single BP reading.

For each participant, 24-h average, daytime (awake) average, and nighttime (asleep) average systolic BP (SBP) and diastolic BP (DBP) were calculated separately for both the reference ABPM device and Alysis-001 under each data extraction strategy. Awake and asleep periods were defined based on participant-reported in-bed and out-of-bed times.

Awake-to-asleep BP change (Δ BP) was calculated for each participant as:

Δ BP = (Awake BP) − (Asleep BP)

This calculation was performed separately for SBP and DBP, for both the reference ABPM device and Alysis-001 under each data extraction strategy.

#### Dipping pattern classification

Participants were classified into dipping categories based on the percentage decrease in nocturnal BP relative to daytime BP, calculated as:

Nocturnal BP dipping (%) = [(Awake BP–Asleep BP)/Awake BP] × 100

Categories were defined as:

- Extreme dipper: ≥20% decrease
- Dipper: 10–19% decrease
- Non-dipper: 0–9% decrease
- Reverse dipper: Increase in nocturnal BP (negative value)

Dipping classification was performed separately using reference ABPM and Alysis-001 data under each data extraction strategy.

#### Data management and blinding

To ensure data integrity and minimize potential bias, data collection, processing, and analysis were conducted by independent research teams with appropriate blinding procedures.

Measurements were performed by the Department of Geriatric and General Medicine, the University of Osaka. Alysis-001 BP estimates were processed by Arblet Inc., who exported the data without participant identifiers. Reference ABPM data and de-identified Alysis-001 data were provided separately to the Division of Public Health, the University of Osaka, without any linking information. The Division of Public Health subsequently matched participant identifiers between the two datasets and performed all statistical analyses. This approach prevented the algorithm development team from accessing reference ABPM values during BP estimation processing, ensuring independence between cuffless BP estimation and evaluation analysis.

### Statistical analysis

#### Agreement analysis

Agreement between Alysis-001 and reference ABPM was assessed using:

- Paired t-tests to evaluate mean differences in 24-h, daytime, and nighttime SBP and DBP
- Bland-Altman analysis to visualize agreement and identify systematic bias
- Intraclass correlation coefficients (ICCs) with 95% confidence intervals to quantify consistency between measurements
- Pearson correlation coefficients to assess linear relationships between devices

All analyses were performed separately for each data extraction strategy.

#### ESH awake/asleep test criteria

According to the European Society of Hypertension (ESH) recommendations for cuffless BP device evaluation [8], the primary evaluation endpoint was whether Alysis-001 met the following awake/asleep test criteria under each data extraction strategy:

- Mean difference between awake-asleep BP changes (Alysis-001 vs. reference ABPM) ≤ 5 mm Hg for both SBP and DBP
- Standard deviation (SD) of the difference ≤ 8 mm Hg for both SBP and DBP
- Correlation coefficient between awake-asleep BP changes (Alysis-001 vs. reference ABPM) ≥ 0.70 for both SBP and DBP

These criteria were evaluated using the difference between participant-level awake-asleep BP changes calculated from Alysis-001 and reference ABPM.

#### Diagnostic performance

Sensitivity, specificity, positive predictive value (PPV), negative predictive value (NPV), and overall accuracy of Alysis-001 for identifying non-dippers (nocturnal dipping <10%) were calculated using reference ABPM classification as the gold standard. A 2×2 contingency table was constructed, and diagnostic performance metrics were computed with 95% confidence intervals. These analyses were performed separately for each data extraction strategy.

### Subgroup analysis

To assess the consistency of Alysis-001 accuracy across diverse patient characteristics, subgroup analyses were performed stratified by:

- Sex (male vs. female)
- Age ( < 65 years vs. ≥65 years)
- BMI ( < 25 kg/m² vs. ≥25 kg/m²)
- Antihypertensive medication classes (angiotensin receptor blockers, calcium channel blockers, diuretics, beta-blockers, ACE inhibitors)
- BP status (SBP: ≥130 mmHg. 120–130 mmHg, <120 mmHg, DBP: ≥80 mmHg, 70–80 mmHg, <70 mmHg)

For each subgroup, mean differences, ICCs, and correlation coefficients were calculated separately for SBP and DBP under each data extraction strategy.

All statistical analyses were conducted using R (version 4.4.2; R Foundation for Statistical Computing, Vienna, Austria) and RStudio (version 2025.01.0; RStudio Team, Boston, MA, USA). A two-sided *p*-value < 0.05 was considered statistically significant.

## Results

### Participant flow and characteristics

Of the 92 hypertensive patients who consented and wore both devices, 78 were included in the final analysis after exclusion of four participants who removed the wearable device during monitoring, one participant without nighttime measurements, one participant with missing ABPM data, and eight participants who did not have 12 valid asleep readings (Fig. 1c). All analyzed participants were receiving antihypertensive treatment. Baseline characteristics are summarized in Table 1.

**Table 1:** Patient characteristics

Factors		
Anthropological factors		
Age	years	69.9 ± 13.1
Sex	men (%)	41 (53)
BMI	kg/m^2^	24.1 ± 3.1
Wrist circumference	mm	163.0 ± 12.8
Arm circumference	mm	263.5 ± 26.7
Dominant hand	Right (%)	78 (100)
Circadian blood pressure rhythm		
Daytime hypertension	*n* (%)	37 (47)
Nighttime hypertension	*n* (%)	37 (47)
Medication		
CCB	*n* (%)	61 (78)
ARB/ACEi	*n* (%)	36 (46)
Diuretics	*n* (%)	6 (8)
Beta blocker	*n* (%)	12 (15)
MRA	*n* (%)	19 (24)
ARNi	*n* (%)	15 (19)

Data are presented as mean ± standard deviation or n (%)

*BMI* body mass index, *CCB* calcium channel blocker, *ARB* angiotensin receptor blocker, *ACEi* angiotensin converting enzyme inhibitor, *MRA* mineralocorticoid receptor antagonist, *ARNI* angiotensin receptor neprilysin inhibitor

Main analyses were performed using two data extraction strategies: Strategy 1 (arm-stabilized periods only, ≥10 s static) and Strategy 2 (including low-intensity activity, with high-intensity activity excluded). Results for both strategies are presented together to enable direct comparison.

### Comparison between alysis-001 and conventional ABPM

#### 24-h, awake, and asleep blood pressure values

Mean 24-h, awake, and asleep systolic and diastolic BP values measured by Alysis-001 and conventional ABPM are shown in Table 2 (Strategy 1: Table 2a; Strategy 2: Table 2b). Across all periods and under both data extraction strategies, mean differences between the two methods were within the acceptable range ( ≤ 5 mmHg) with standard deviations ≤8 mmHg. The number of BP readings used for the calculation of awake, sleep, and 24-h averages was similar between ABPM and the wearable device (Supplementary Table 2).

**Table 2:** Mean 24-h, daytime, and night-time BP as obtained by Alysis and conventional ABPM (*N* = 78). a. strategy 1 (arm-stabilized periods only, ≥ 10 s static). b. strategy 2 (including low-intensity activity, with high-intensity activity excluded)

a				
	Alysis	ABPM	Difference	*P* value
Systolic BP(mmHg)				
24-h	124.91 ± 9.56	126.05 ± 10.76	–1.14 [–2.04, –0.23]	0.002
Awake	128.91 ± 9.98	131.28 ± 11.33	–2.37 [–3.43,–1.30]	<0.001
asleep	116.33 ± 12.72	114.52 ± 14.04	1.81 [0.92, 2.70]	<0.001
awake-sleep difference	12.59 ± 11.09	16.76 ± 13.12	–4.18 [–5.37, –2.98]	<0.001
Diastolic BP(mmHg)				
24-h	75.09 ± 6.32	76.43 ± 7.12	–1.34 [–1.91, –0.78]	<0.001
awake	77.77 ± 6.55	80.04 ± 7.55	–2.27 [–3.00, –1.54]	<0.001
asleep	69.32 ± 8.04	68.49 ± 8.55	0.83 [0.41, 1.25]	<0.001
awake-sleep difference	8.45 ± 6.86	11.55 ± 7.87	–3.10 [–3.91, –2.29]	<0.001
Nocturnal dip				
dipping (%)	10.7 ± 8.5	14.2 ± 9.2	–3.5 [–4.4, –2.6]	<0.001

b				
	Alysis	ABPM	Difference	*P* value
Systolic BP(mmHg)				
24-h	125.80 ± 9.02	126.05 ± 10.76	–0.25 [–1.04, 0.55]	0.535
awake	129.78 ± 9.20	131.28 ± 11.33	–1.50 [–2.38, –0.62]	0.001
asleep	116.87 ± 12.54	114.52 ± 14.04	2.36 [1.40, 3.32]	<0.001
awake-sleep difference	12.91 ± 10.77	16.76 ± 13.12	–3.86 [–5.02, –2.69]	<0.001
Diastolic BP(mmHg)				
24-h	76.14 ± 6.12	76.43 ± 7.12	–0.29 [–0.79, 0.21]	0.249
awake	78.95 ± 6.28	80.04 ± 7.55	–1.09 [–1.72, –0.47]	<0.001
asleep	69.86 ± 7.88	68.49 ± 8.55	1.37 [0.83, 1.91]	<0.001
awake-sleep difference	9.09 ± 6.68	11.55 ± 7.87	–2.46 [–3.26, –1.66]	<0.001
Nocturnal dip				
dipping (%)	9.9 ± 8.0	12.6 ± 9.3	–2.7 [–3.5, –1.9]	<0.001

Data are presented as mean ± standard deviation

*BP* blood pressure, *ABPM* ambulatory blood pressure monitoring

Dipping (%) was calculated as (awake BP − asleep BP) / awake BP × 100

Compared with ABPM, Alysis-001 tended to yield lower awake BP values and slightly higher asleep BP values under both strategies. Consequently, the calculated nocturnal BP dipping rate was smaller with Alysis-001 than with ABPM.

Inclusion of low-intensity activity measurements (Strategy 2) resulted in modest increases in both awake and asleep BP values compared with arm-stabilized periods only (Strategy 1). For Strategy 1, awake SBP was 128.91 ± 9.98 mmHg and asleep SBP was 116.33 ± 12.72 mmHg; for Strategy 2, these values increased to 129.78 ± 9.20 mmHg (awake) and 116.87 ± 12.54 mmHg (asleep). Similar patterns were observed for DBP. Despite these increases, both strategies maintained high agreement with ABPM, and the magnitude of mean differences remained within clinically acceptable limits.

#### Agreement and correlation

**Fig. 2:**
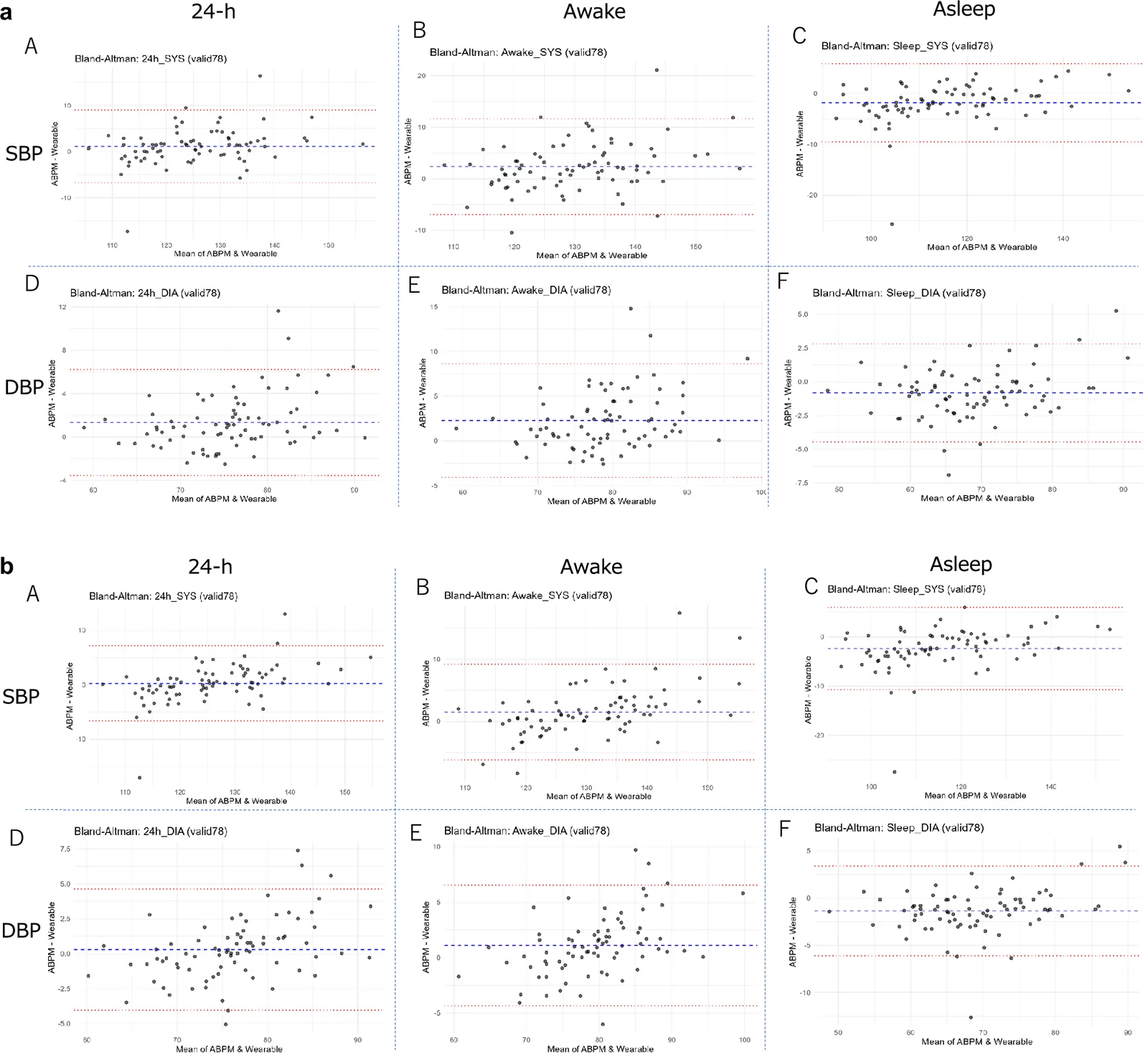
Bland-Altman plot comparing the average 24-h, daytime and night-time BP between Alysis and conventional ABPM analyzed by **a** strategy 1 (arm-stabilized periods only, ≥10 s static) and **b** strategy 2 (including low-intensity activity, with high-intensity activity excluded). Bland-Altman plot of the comparison of the 24-h systolic BP is shown in panel A, awake systolic BP in panel B, asleep systolic in **C**, 24-h diastolic BP in **D**, awake diastolic BP in **E**, asleep diastolic BP in **F**

**Fig. 3:**
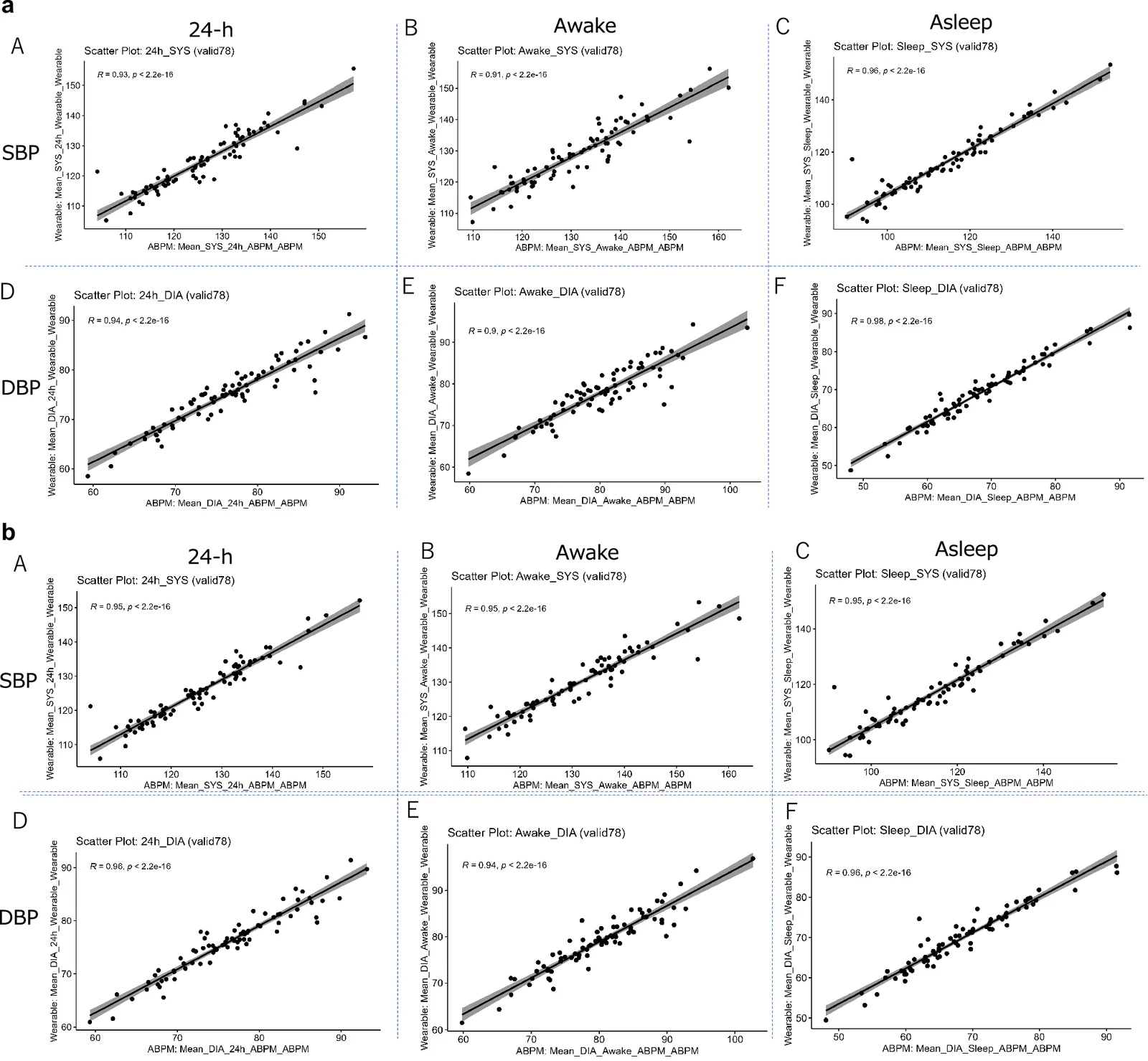
Scatter plot of the average 24-h, daytime and night-time BP between Alysis and conventional ABPM analyzed by **a** strategy 1 (arm-stabilized periods only, ≥10 s static) and **b** strategy 2 (including low-intensity activity, with high-intensity activity excluded). Scatter plot of the 24-h systolic BP is shown in **A**, awake systolic BP in **B**, asleep systolic in **C**, 24-h diastolic BP in **D**, awake diastolic BP in **E**, asleep diastolic BP in **F**

Bland–Altman plots demonstrated good agreement between Alysis-001 and ABPM for 24-h, awake, and asleep systolic and diastolic BP, with no evidence of systematic proportional bias (Fig. 2: Strategy 1–Fig. 2a; Strategy 2–Fig. 2b). Scatter plots showed strong linear correlations across all periods (Fig. 3: Strategy 1–Fig. 3a; Strategy 2–Fig. 3b).

Intraclass correlation coefficients exceeded 0.70 for systolic and diastolic BP in all time periods under both data extraction strategies, indicating reliable agreement between the two measurement methods (Table 3: Strategy 1–Table 3a; Strategy 2– Table 3b). Notably, ICCs under Strategy 2 were comparable to or slightly higher than those under Strategy 1 (e.g., 24-h SBP ICC: 0.928 for Strategy 1 vs. 0.952 for Strategy 2), suggesting that inclusion of low-intensity activity measurements did not compromise measurement reliability.

**Table 3:** Summary of the agreement statistics between Alysis and conventional ABPM. a. strategy 1 (arm-stabilized periods only, ≥ 10 s static). b. Strategy 2 (including low-intensity activity, with high-intensity activity excluded)

a							
Metric	*N*	Pearson_r	Mean_Diff	SD_Diff	ICC	ICC_Lower	ICC_Upper
24h-SBP	78	0.928	1.14	4.03	0.917	0.869	0.947
24h-DBP	78	0.938	1.34	2.5	0.914	0.815	0.954
Awake-SBP	78	0.909	2.37	4.73	0.881	0.767	0.934
Awake-DBP	78	0.904	2.27	3.24	0.852	0.605	0.93
Sleep-SBP	78	0.962	–1.81	3.93	0.949	0.901	0.971
Sleep-DBP	78	0.977	–0.83	1.86	0.97	0.943	0.983

b							
Metric	*N*	Pearson_r	Mean_Diff	SD_Diff	ICC	ICC_Lower	ICC_Upper
24h-SBP	78	0.952	0.25	3.52	0.938	0.904	0.96
24h-DBP	78	0.955	0.29	2.21	0.944	0.914	0.964
Awake-SBP	78	0.948	1.5	3.92	0.919	0.861	0.951
Awake-DBP	78	0.936	1.09	2.77	0.91	0.844	0.946
Sleep-SBP	78	0.955	–2.36	4.27	0.934	0.852	0.966
Sleep-DBP	78	0.96	–1.37	2.41	0.944	0.869	0.972

*BP* blood pressure, *ABPM* ambulatory blood pressure monitoring, *SBP* systolic blood pressure, *DBP* diastolic blood pressure, *Diff* difference, *SD* standard deviation, *ICC* Intraclass Correlation Coefficient

**Fig. 4:**
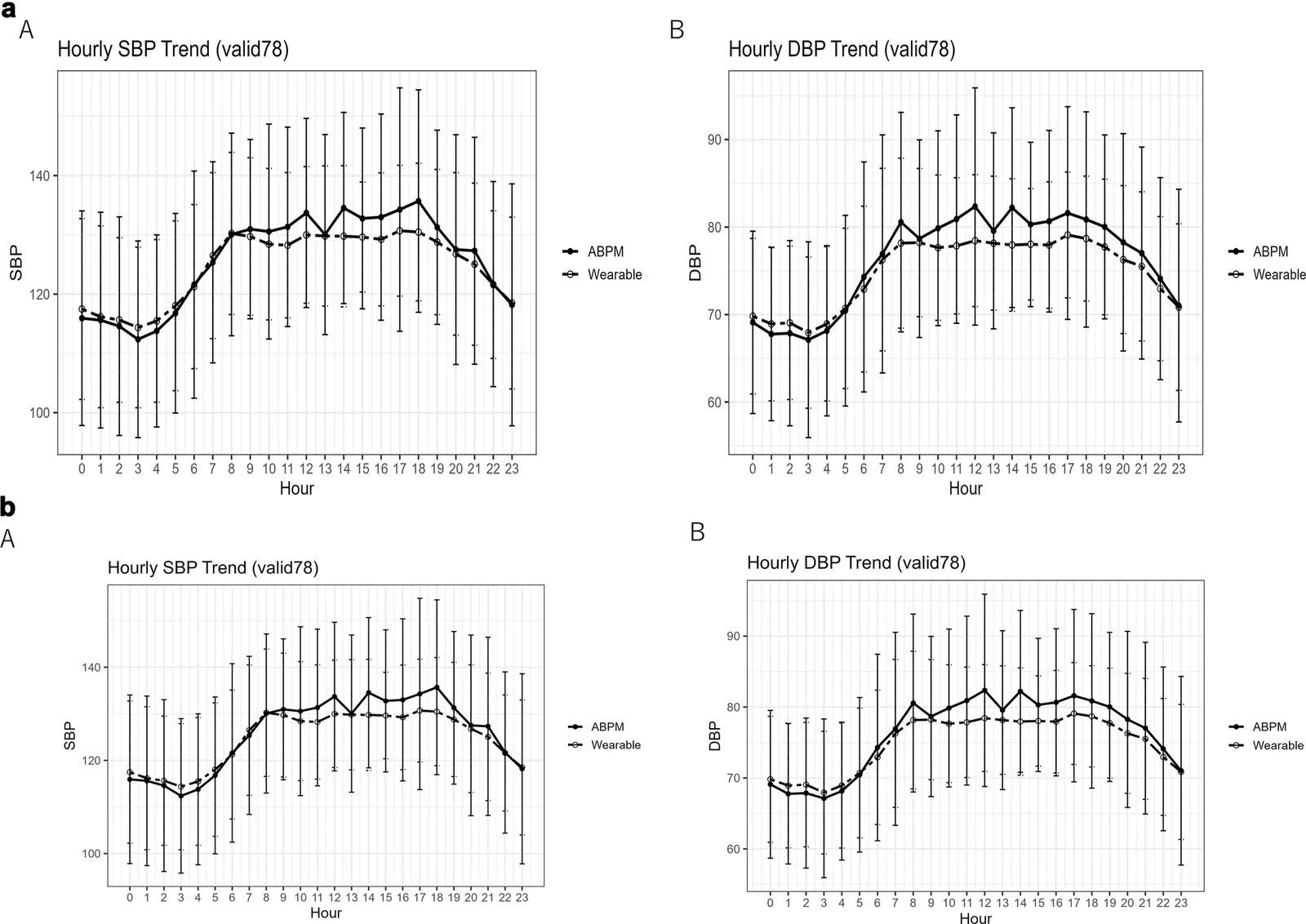
Hourly mean ± SD systolic and diastolic blood pressure profiles measured by Alysis and conventional ABPM analyzed by **a** strategy 1 (arm-stabilized periods only, ≥10 s static) and **b** strategy 2 (including low-intensity activity, with high-intensity activity excluded). A shows hourly mean ± SD systolic blood pressure profiles, and B shows hourly mean ± SD diastolic blood pressure profiles measured by the devices

Hourly BP profiles further demonstrated close tracking of diurnal BP variation by Alysis-001, with small mean differences throughout the 24-h period under both strategies. However, awake BP values were consistently lower with Alysis-001 than with ABPM (Fig. 4: Strategy 1–Fig. 4a; Strategy 2–Fig. 4b).

### Evaluation against the ESH awake/asleep test criteria

#### Awake–asleep blood pressure change

**Fig. 5:**
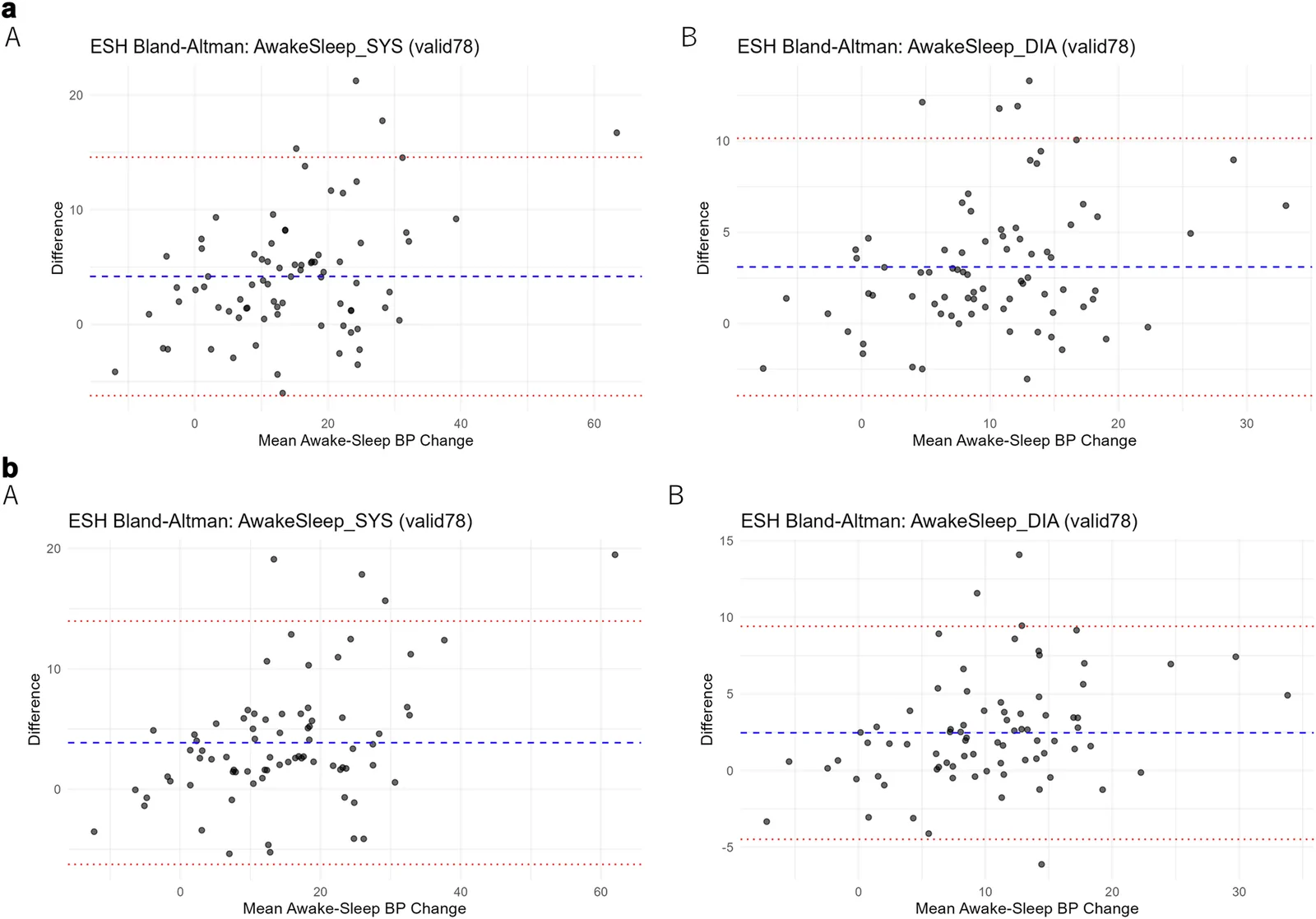
Bland-Altman plot comparing the awake-sleep BP change between Alysis and conventional ABPM analyzed by **a** strategy 1 (arm-stabilized periods only, ≥10 s static) and **b** strategy 2 (including low-intensity activity, with high-intensity activity excluded). Bland-Altman plot of the awake-sleep systolic BP change is shown in **A**, awake-sleep diastolic BP change in **B**

**Fig. 6:**
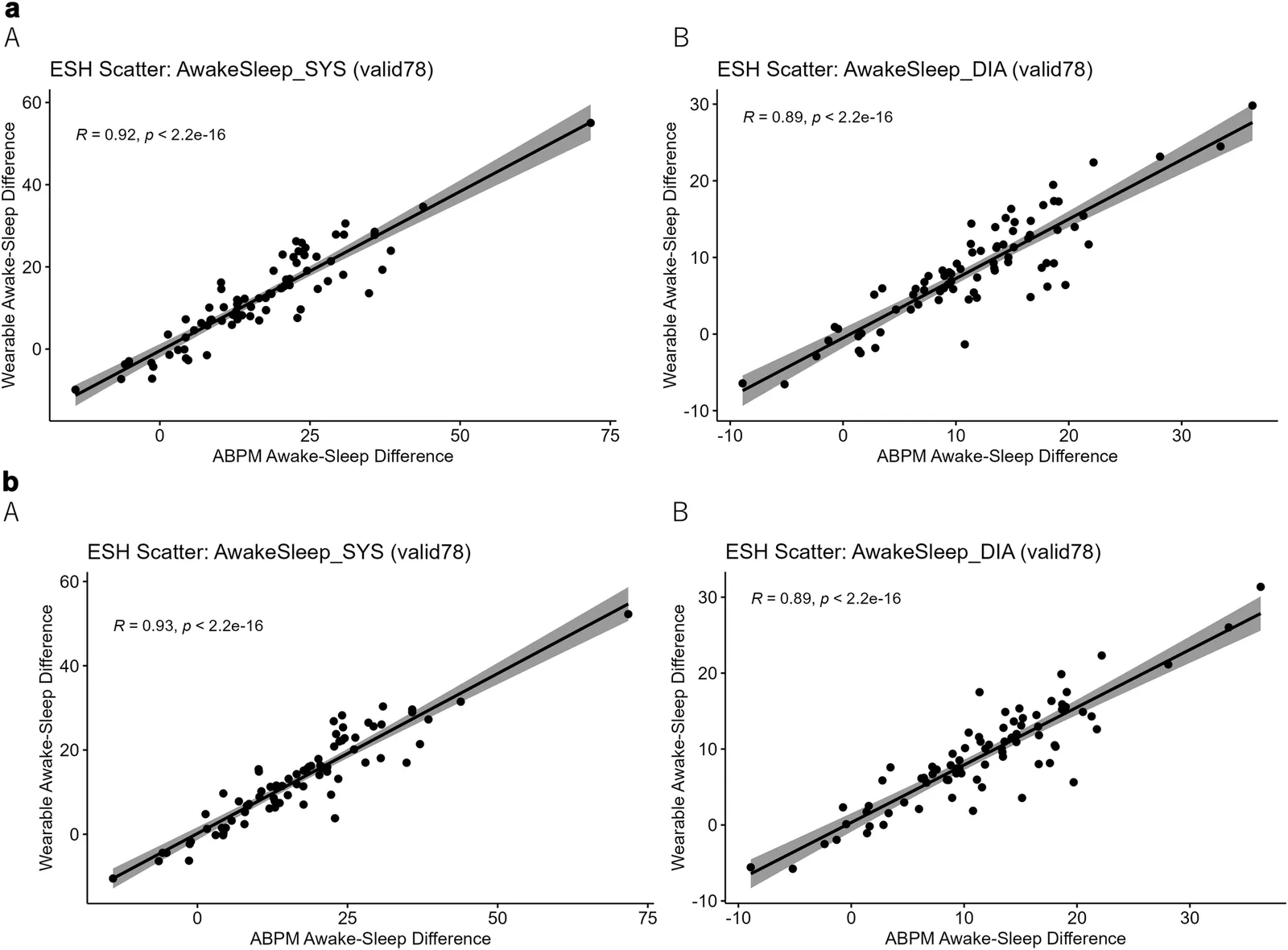
Scatter plot of the awake-sleep BP change between Alysis and conventional ABPM, analyzed by **a** strategy 1 (arm-stabilized periods only, ≥10 s static) and **b** strategy 2 (including low-intensity activity, with high-intensity activity excluded). Scatter plot of the awake-sleep systolic BP change is shown in **A**, awake-sleep diastolic BP change in **B**

Agreement for awake–asleep BP changes between Alysis-001 and ABPM is illustrated by Bland–Altman and scatter plots (Figs. 5 and 6; Strategy 1: Figs. 5a, 6a; Strategy 2: Figs. 5b, 6b). The distributions showed close clustering around zero difference for both systolic and diastolic BP changes under both data extraction strategies.

#### Fulfillment of ESH evaluation criteria

Summary statistics for awake–asleep BP change are presented in Table 4 (Strategy 1: Table 4a; Strategy 2: Table 4b). Alysis-001 satisfied all ESH awake/asleep test criteria under both data extraction strategies:

- Mean difference between awake–asleep BP changes ≤5 mmHg (Strategy 1: SBP 4.18 mmHg, DBP 3.10 mmHg; Strategy 2: SBP 3.86 mmHg, DBP 2.46 mmHg)
- Standard deviation ≤8 mmHg (Strategy 1: SBP 5.31 mmHg, DBP 3.60 mmHg; Strategy 2: SBP 5.16 mmHg, DBP 3.54 mmHg)
- Correlation coefficient ≥0.70 (Strategy 1: SBP r = 0.917, DBP r = 0.890; Strategy 2: SBP r = 0.925, DBP r = 0.894)

**Table 4:** Summary of the agreement statistics of awake-asleep BP change between Alysis and conventional ABPM. a. strategy 1 (arm-stabilized periods only, ≥10 s static). b. Strategy 2 (including low-intensity activity, with high-intensity activity excluded)

a							
Metric	*N*	Pearson_r	Mean_Diff	SD_Diff	ICC	ICC_Lower	ICC_Upper
Awake-Sleep SBP change	78	0.917	4.18	5.31	0.855	0.544	0.937
Awake-Sleep DBP change	78	0.89	3.1	3.6	0.811	0.391	0.92

b							
Metric	*N*	Pearson_r	Mean_Diff	SD_Diff	ICC	ICC_Lower	ICC_Upper
Awake-Sleep SBP change	78	0.925	3.86	5.16	0.864	0.595	0.939
Awake-Sleep DBP change	78	0.894	2.46	3.54	0.836	0.576	0.922

*BP* blood pressure, *ABPM* ambulatory blood pressure monitoring, *SBP* systolic blood pressure, *DBP* diastolic blood pressure, *Diff* difference, *SD* standard deviation, *ICC* Intraclass Correlation Coefficient

These criteria were met for both systolic and diastolic BP under both Strategy 1 and Strategy 2. ICC values also exceeded 0.70 for both strategies (Strategy 1: SBP ICC = 0.855, DBP ICC = 0.811; Strategy 2: SBP ICC = 0.864, DBP ICC = 0.836), demonstrating that inclusion of low-intensity activity measurements maintained compliance with ESH evaluation standards while preserving measurement reliability.

#### Dipping pattern classification

Cross-tabulation of dipping categories is shown in Table 5 (Strategy 1: Table 5a; Strategy 2: Table 5b). Using ABPM as the reference, Alysis-001 demonstrated high sensitivity (0.93 for both strategies), specificity (0.75 for both strategies), and overall accuracy (0.82 for both strategies) for identifying non-dippers (Table 6: Strategy 1–Table 6a; Strategy 2–Table 6b).

**Table 5:** Cross-table of dipping category between Alysis and conventional ABPM. a. strategy 1 (arm-stabilized periods only, ≥ 10 s static). b. strategy 2 (including low-intensity activity, with high-intensity activity excluded)

a					
		Alysis			
ABPM		extreme dipper	dipper	non-dipper	Total
	extreme dipper	6	8	1	15
	dipper	1	21	11	33
	non-dipper	0	2	28	30
	Total	7	31	40	78

b					
		Alysis			
ABPM		extreme dipper	dipper	non-dipper	Total
	extreme dipper	8	6	1	15
	dipper	2	20	11	33
	non-dipper	0	2	28	30
	Total	10	28	40	78

*BP* blood pressure, *ABPM* ambulatory blood pressure monitoring

**Table 6:** diagnostic performance of Alysis-based classification in identifying non-dippers compared to conventional ABPM. a. strategy 1 (arm-stabilized periods only, ≥ 10 s static). b. strategy 2 (including low-intensity activity, with high-intensity activity excluded)

a	
Metric	Value
Sensitivity	0.93
Specificity	0.75
Accuracy	0.82

Sensitivity	those classified as non-dippers by ABPM who were also classified as non-dippers by Alysis
Specificity	those classified as not non-dippers by ABPM who were also classified as not non-dippers by Alysis
Accuracy	those with matching classifications between ABPM and Alysis

b	
Metric	Value
Sensitivity	0.93
Specificity	0.75
Accuracy	0.82

Sensitivity	those classified as non-dippers by ABPM who were also classified as non-dippers by Alysis
Specificity	those classified as not non-dippers by ABPM who were also classified as not non-dippers by Alysis
Accuracy	those with matching classifications between ABPM and Alysis

*BP* blood pressure, *ABPM* ambulatory blood pressure monitoring

Nevertheless, systematic shifts in dipping classification were observed under both strategies. Participants classified as extreme dippers by ABPM were more frequently classified as dippers by Alysis-001, while ABPM-defined dippers tended to be classified as non-dippers. This shift was primarily attributable to lower awake BP values measured by Alysis-001, resulting in smaller awake–asleep BP differences. Inclusion of low-intensity activity measurements (Strategy 2) did not substantially alter these classification patterns compared with Strategy 1, as both awake and asleep BP increased proportionally, maintaining similar awake-asleep differences.

#### Subgroup analyses

Subgroup analyses were performed using Strategy 1 (arm-stabilized periods only). Analyses stratified by sex (supplementary tables 3–4), age tertiles (supplementary tables 5–6), BMI tertiles (supplementary tables 7–8), medication classes (supplementary tables 9–20), daytime hypertension (supplementary tables 21–22), nighttime hypertension (supplementary tables 23–24), BP classification (supplementary tables 25–36) demonstrated consistent performance of Alysis-001. Across all subgroups, mean differences, standard deviations, and correlation coefficients for awake–asleep BP changes showed no clinically meaningful variation, supporting robustness of the algorithm across diverse patient characteristics.

## Discussion

This study evaluated Alysis-001, a BP estimation software algorithm (SaMD) operating based on sensor data acquired with a wearable device, in a cohort of hypertensive patients receiving antihypertensive treatment. Analyses were performed using two data extraction strategies: Strategy 1 (arm-stabilized periods only, ≥10 s static) and Strategy 2 (including low-intensity activity, with high-intensity activity excluded).

### Key findings

First, Alysis-001 demonstrated excellent agreement with reference ABPM across all periods, with ICC > 0.7 under both data extraction strategies. Across 24-h, daytime, and nighttime measurements, mean differences remained within acceptable limits and ICCs remained high for both strategies, indicating robust measurement reliability regardless of whether low-intensity activity periods were included.

Second, Alysis-001 showed high diagnostic performance for identifying non-dippers under both strategies (sensitivity 0.93 for both; specificity 0.75 for both strategies; accuracy 0.82 for both strategies). However, Alysis-001 estimated smaller awake-asleep BP differences than ABPM, causing classification shifts (ABPM extreme dippers→Alysis-001 dippers; ABPM dippers→Alysis-001 non-dippers). This pattern was observed under both data extraction strategies, appearing related to lower daytime and modestly higher values in Alysis-001. Notably, inclusion of low-intensity activity measurements (Strategy 2) resulted in modest increases in both awake BP (128.91 to 129.78 mmHg for SBP) and asleep BP (116.33 to 116.87 mmHg for SBP), representing proportional increases that maintained similar awake-asleep differences between strategies. Consequently, dipping classification patterns remained consistent between the two approaches.

Most importantly, Alysis-001 successfully passed the ESH awake/asleep test in this hypertensive cohort, meeting all criteria (mean difference ≤5 mmHg, SD ≤ 8 mmHg, correlation ≥0.70 for SBP and DBP for both strategies). This test assesses the ability to track clinically important sleep-related BP changes while indirectly evaluating accuracy under varying positions, activities, and motion [7]. The consistency of ESH criteria fulfillment across both strategies demonstrates the robustness of the Alysis-001 algorithm’s ability to track diurnal BP variation.

Although some statistically significant differences between the cuffless device and ABPM were observed, these differences were small in magnitude and remained within the predefined ESH acceptance criteria. This finding highlights the distinction between statistical significance and clinical relevance in the context of device evaluation. Given the relatively large number of paired observations derived from continuous or high-frequency measurements, even minor differences can reach statistical significance. Therefore, statistical significance alone should not be interpreted as evidence of clinically meaningful disagreement. In accordance with the ESH recommendations, the performance of the cuffless device should be primarily evaluated based on agreement metrics, including the mean difference and standard deviation, as well as its ability to capture awake–sleep BP changes. From this perspective, the observed differences are considered acceptable and do not indicate a limitation in device performance. This consideration is particularly important when interpreting results from cuffless devices that rely on continuous signal acquisition.

Stratified analyses performed using Strategy 1 demonstrated consistent accuracy across diverse subgroups, including patients receiving various antihypertensive medication classes, supporting generalizability to real-world hypertensive populations.

### Comparison of data extraction strategies

This study compared two approaches to defining valid measurement periods: restricting BP estimation to arm-stabilized periods only (Strategy 1) versus including both resting and low-intensity activity periods (Strategy 2). While conventional cuff-based ABPM measures BP at predetermined intervals regardless of activity state, cuffless devices must balance measurement reliability with real-world applicability.

Inclusion of low-intensity activity measurements resulted in modest but consistent increases in both awake and asleep BP values. For 24-h measurements, mean differences with ABPM improved from –1.14 mmHg (Strategy 1) to –0.25 mmHg (Strategy 2) for SBP, reflecting closer alignment with reference ABPM. Similarly, ICCs showed slight improvements under Strategy 2 (e.g., 24-h SBP ICC: 0.917 for Strategy 1 vs. 0.938 for Strategy 2), suggesting that inclusion of low-intensity activity data did not compromise measurement reliability.

Critically, both strategies yielded proportional increases in awake and asleep BP, maintaining similar awake-asleep differences and dipping classification patterns. This consistency indicates that the algorithm’s ability to track diurnal BP variation remains robust across different activity inclusion criteria. The fact that both approaches met all ESH awake/asleep test criteria demonstrates that Alysis-001 can maintain clinical validity under varying data extraction strategies, providing flexibility for real-world deployment.

### Algorithm classification and evaluation framework

ESH recommendations classify intermittent cuffless devices into nine types by function, sensor characteristics, and calibration method [7]. Although developed for physical devices, these evaluation principles apply equally to SaMD, as clinical performance requirements are independent of device classification.

Alysis-001 was trained on independent datasets (953 seated-rest, 150 treadmill-walking recordings) with an aneroid sphygmomanometer and ABPM reference values, respectively. Alysis-001, utilizing sensor data acquired with a wrist-worn wearable device platform, corresponds to Device Type 3 cuffless BP device: automated wearable device with demographic calibration and non-heart-level sensor [7]. The algorithm uses age, height, and weight for personalized estimation without initial or periodic cuff calibration.

For Device Type 3 cuffless BP devices, ESH specifies five evaluation tests: static, device position, treatment, awake/asleep, and exercise tests. The awake/asleep test is the “primary test” performed first [7]; only after passing should additional tests proceed.

To our knowledge, this is the first report of successful ESH awake/asleep test completion for a Device Type 3 cuffless BP devices estimation algorithm in a hypertensive patient cohort, representing an important milestone for software-based cuffless monitoring systems using demographic calibration without recalibration requirements.

### Cuffless BP evaluation challenges

Several cuffless ABPM systems have been developed. Aktiia received CE mark certification in 2021 and is marketed in Switzerland, the UK, and France [9]. CART-1 Plus and other systems have also been evaluated [10, 11].

The evaluation landscape has been marked by significant challenges [6]. The Microsoft Research Aurora Project comprehensively evaluated cuffless technologies [8]. When waveform-based models (tonometry+ECG, PPG + ECG, PPG alone) were compared with baseline models using only demographic data without hemodynamic measurements, results showed no significant improvement with waveform features, revealing fundamental limitations in approaches being developed [12].

Aurora Project findings underscored the need for rigorous evaluation procedures, distinguishing truly accurate systems from those failing to outperform simple baseline models. In response, ESH published comprehensive recommendations in 2023 [7]. The ESH framework requires demonstrating accurate BP change tracking—addressing core limitations revealed by Aurora, where many systems failed to provide meaningful improvements over baseline models.

ESH evaluation framework establishment has made it essential for any cuffless system to demonstrate compliance with these rigorous standards to gain clinical credibility. Meeting ESH criteria represents a critical step toward establishing cuffless monitoring as a reliable alternative.

### Clinical significance

Alysis-001 successfully passed the ESH awake/asleep test for Device Type 3 cuffless BP devices in hypertensive patients under both data extraction strategies [7]. This achievement is particularly significant given the Aurora Project challenges and high ESH standards. By demonstrating accurate awake-to-asleep BP change tracking in patients receiving antihypertensive treatment, Alysis-001 represents an important step toward clinical credibility for software-based cuffless monitoring [6].

The awake/asleep test is the most clinically meaningful evaluation because it evaluates diurnal BP tracking essential for hypertension management and cardiovascular risk assessment. Accurately capturing awake-to-asleep decreases is particularly important, as nocturnal patterns predict cardiovascular events [13–15].

Alysis-001 showed consistently high accuracy for identifying dippers and non-dippers among hypertensive patients, with excellent agreement across all time periods. Although smaller awake-asleep differences than ABPM caused some classification shifts, all ESH criteria were met under both strategies.

The robust sample (78 hypertensive patients) with diverse characteristics, including various antihypertensive medication regimens, strengthens generalizability to clinical hypertensive populations. Using 3-axis accelerometer data to classify activity levels into resting, low-intensity, and high-intensity categories demonstrates the capability to optimize measurement accuracy during daily activities. These findings suggest Alysis-001 may identify hypertensive patients at elevated cardiovascular risk without cuff-based ABPM discomfort.

### Software as a medical device considerations

The distinction between validating physical devices versus SaMD is primarily regulatory rather than scientific. ESH testing procedures assess functional performance and accuracy identically, as clinical endpoints (measurement accuracy, change tracking, position robustness) are independent of device classification.

However, SaMD presents unique considerations beyond traditional evaluation: algorithm versioning, software updates, cybersecurity, and platform compatibility [16]. While this study validates Alysis-001 clinical accuracy on a specific platform in a hypertensive population, ongoing quality assurance and post-market surveillance will ensure continued accuracy across updates and potential future platforms.

### Limitations

First, regarding BP distribution, ESH recommends ≥30% of subjects have 24-h DBP < 70 mmHg [7]. In the present study, the proportion meeting this criterion was <30%, while all other ESH-recommended BP distribution criteria had more than 30% of the population; however, the absolute number (*n* = 16) exceeded the minimum requirement (35 × 30% = 10.5). When stratified into three categories (SBP: ≥130, 120-130, <120 mmHg; DBP: ≥80, 70-80, <70 mmHg), measurement errors showed no substantial variation, suggesting consistent accuracy across BP ranges within this treated hypertensive population (supplementary table 23-34).

Second, this study evaluated two data extraction strategies. Strategy 1 (arm-stabilized only) restricts analysis to periods of minimal motion artifacts, while Strategy 2 (including low-intensity activity) provides broader coverage of daily activities. Both strategies demonstrated comparable performance and met all ESH criteria. However, both approaches showed that Alysis-001 yielded lower daytime BP values and slightly higher nighttime BP values compared with ABPM, resulting in smaller awake-asleep differences and some dipping classification shifts (ABPM extreme dippers→Alysis-001 dippers; ABPM dippers→Alysis-001 non-dippers). Clinicians should be aware of these observed differences between cuff-based ABPM and Alysis-001 when interpreting dipping patterns in clinical practice.

Third, this study reports only the awake/asleep test (primary test for Device Type 3 cuffless BP devices) in a hypertensive cohort. Additional ESH tests are required for comprehensive evaluation and will be reported subsequently.

In addition, all participants in this study were hypertensive patients receiving antihypertensive treatment. While this design reflects real-world clinical practice, where most patients with hypertension are treated, it may limit the generalizability of our findings to untreated hypertensive individuals or normotensive populations. Antihypertensive medications could potentially influence blood pressure variability and diurnal patterns, which may in turn affect the performance of cuffless blood pressure estimation algorithms. Therefore, further validation studies including untreated and normotensive populations are warranted to confirm the broader applicability of Alysis-001.

Lastly, Recent studies have questioned the rationale and practical value of the ESH awake/asleep test, noting that even reference ABPM devices may not consistently satisfy its criteria [17]. These findings suggest that the test may represent a stringent and still-evolving evaluation framework. In this context, the fact that Alysis-001 fulfilled the ESH criteria should be interpreted with caution. The observed performance may partly reflect differences in measurement characteristics, such as the higher temporal resolution of cuffless continuous monitoring. Therefore, evaluation results should be interpreted not only based on predefined criteria but also in relation to the underlying measurement principles.

### Future perspectives

Successful awake/asleep test completion in this hypertensive cohort under multiple data extraction strategies represents a crucial first step in comprehensive Alysis-001 evaluation. Further studies are planned to assess performance across diverse clinical scenarios, including normotensive populations and various disease states. Through continued development and evaluation, we aim to establish a software-based cuffless ABPM system achieving medical-grade accuracy for clinical hypertension diagnosis and management. By meeting rigorous standards, we hope to contribute to building clinical trust for cuffless monitoring technologies, ultimately providing patients with a more comfortable alternative for long-term BP monitoring.

## Supplementary information

Supplementary Information

## Data Availability

The independent training datasets used for the development of the Alysis-001 algorithm are not publicly available due to strict ethical restrictions and patient data privacy regulations regarding the original cohorts. The clinical evaluation datasets analyzed during the current study are available from the corresponding author on reasonable request, subject to institutional ethical approval.
