# Supplementary material for: Evaluation of Alysis-001 cuffless blood pressure estimation algorithm against the European Society of Hypertension awake/asleep test criteria in hypertensive patients
Source: Hypertens Res. 2026 Aug 21;49(11):3137–53. doi: 10.1038/s41440-026-02728-z (PMC13736482; doi:10.1038/s41440-026-02728-z)
Supplement: Supplementary file 1 [file 41440_2026_2728_MOESM1_ESM.docx]

**Supplementary Table 1: Baseline characteristics of the independent training datasets**

|  | **Mean** | **SD** | **Range** |
| --- | --- | --- | --- |
| Age (years) | 51.21 | 14.67 | 18 – 90 |
| Height (cm) | 164.26 | 8.63 | 141.00 – 189.10 |
| Weight (kg) | 66.36 | 14.68 | 36.40 – 141.60 |
| BMI (kg⸱m^-2^) | 24.47 | 4.45 | 15.15 – 51.51 |
| Wrist Size (mm) | 159.41 | 14.39 | 130.00 – 208.00 |
| UAC (mm) | 263.41 | 33.06 | 196.00 – 390.00 |
| SBP (mm Hg) | 117.74 | 23.39 | 68.29 – 209.57 |
| DBP (mm Hg) | 75.04 | 15.44 | 33.14 – 139.29 |

BMI, body mass index; UAC, upper arm circumference; SBP, systolic blood pressure; DBP, diastolic blood pressure; SD, standard deviation.

**Supplementary Table 2. Number of blood pressure readings used for the calculation of awake, sleep, and 24-hour averages for Alysis and conventional ABPM**

| Method | Period | Mean ± SD | Range |
| --- | --- | --- | --- |
| Alysis-001 | 24h | 49.1 ± 2.8 | 36–53 |
| Alysis-001 | Awake | 33.1 ± 3.6 | 25–39 |
| Alysis-001 | Sleep | 15.9 ± 2.6 | 12–23 |
| Conventional ABPM | 24h | 49.1 ± 1.9 | 40–53 |
| Conventional ABPM | Awake | 33.1 ± 3.2 | 26–39 |
| Conventional ABPM | Sleep | 15.9 ± 2.6 | 12–23 |

**Supplementary table 3 mean 24-h, daytime, and night-time BP as obtained by Alysis and conventional ABPM stratified by sex (N=86)**

|  | **Men** | | | **Women** | | |
| --- | --- | --- | --- | --- | --- | --- |
|  | **Alysis** | **ABPM** | **P value** | **Alysis** | **ABPM** | **P value** |
| **Systolic BP (mmHg)** | | | |  |  |  |
| **24-h** | **128.02 ± 10.43** | **128.39 ±11.74** | **0.557** | **121.47 ± 7.17** | **123.46 ± 9.02** | **0.005** |
| **Awake** | **131.82 ± 10.11** | **133.43 ± 11.19** | **0.018** | **125.70 ± 8.89** | **128.91 ± 11.14** | **<0.001** |
| **Asleep** | **120.54 ± 13.98** | **118.27 ± 15.82** | **0.003** | **111.65 ± 9.28** | **110.36 ± 10.48** | **0.01** |
| **Diastolic BP (mmHg)** | | | |  |  |  |
| **24-h** | **77.02 ± 6.32** | **77.80 ± 7.33** | **0.031** | **72.95 ± 5.67** | **74.91 ± 6.66** | **<0.001** |
| **Awake** | **79.78 ± 6.62** | **81.44 ± 7.87** | **<0.001** | **75.55 ± 5.79** | **78.49 ± 6.96** | **<0.001** |
| **Asleep** | **71.49 ± 7.71** | **70.43 ± 8.48** | **0.001** | **66.91 ± 7.82** | **66.35 ± 8.21** | **0.053** |
| **Nocturnal Dip** | | | |  |  |  |
| **Dipping (%)** | **8.6 ± 7.7** | **11.4 ± 8.8** | **<0.001** | **10.8 ± 8.7** | **13.9 ± 9.7** | **<0.001** |

**BP, blood pressure; ABPM, ambulatory blood pressure monitoring**

**Dipping (%) was calculated as (awake BP − asleep BP) / awake BP × 100.**

**Supplementary table 4 summary of the agreement statistics of awake-asleep BP change between Alysis and conventional cuff ABPM stratified by sex**

**Men**

| **Metric** | **N** | **Pearson_r** | **Mean_Diff** | **SD_Diff** | **ICC** | **ICC_Lower** | **ICC_Upper** |
| --- | --- | --- | --- | --- | --- | --- | --- |
| **Awake-Sleep SBP change** | **41** | **0.921** | **3.88** | **4.68** | **0.86** | **0.5** | **0.945** |
| **Awake-Sleep DBP change** | **41** | **0.911** | **2.73** | **3.27** | **0.839** | **0.449** | **0.937** |

**Women**

| **Metric** | **N** | **Pearson_r** | **Mean_Diff** | **SD_Diff** | **ICC** | **ICC_Lower** | **ICC_Upper** |
| --- | --- | --- | --- | --- | --- | --- | --- |
| **Awake-Sleep SBP change** | **37** | **0.912** | **4.5** | **5.97** | **0.851** | **0.535** | **0.939** |
| **Awake-Sleep DBP change** | **37** | **0.871** | **3.51** | **3.94** | **0.788** | **0.301** | **0.918** |

**BP, blood pressure; ABPM, ambulatory blood pressure monitoring; SBP, systolic blood pressure; DBP, diastolic blood pressure; Diff, difference; SD, standard deviation; ICC, Intraclass Correlation Coefficient**

**Supplementary table 5 mean 24-h, daytime, and night-time BP as obtained by Alysis and conventional ABPM stratified by sge tertile (N=78)**

|  | **Age T1 (20-69 y.o.)**  **N=29** | | | **Age T2 (70-77 y.o.)**  **N=29** | | | **Age T3 (78-89 y.o.)**  **N=28** | | |
| --- | --- | --- | --- | --- | --- | --- | --- | --- | --- |
|  | **Alysis** | **ABPM** | **P value** | **Alysis** | **ABPM** | **P value** | **Alysis** | **ABPM** | **P value** |
| **Systolic BP (mmHg)** | | | |  |  |  |  |  |  |
| **24-h** | **124.12 ± 9.87** | **125.18 ± 10.35** | **0.084** | **122.97 ± 7.58** | **124.84 ± 9.27** | **0.029** | **127.65 ± 10.68** | **128.12 ± 12.52** | **0.619** |
| **Awake** | **128.63 ± 10.44** | **130.59 ± 11.21** | **0.029** | **126.73 ± 7.70** | **130.15 ± 9.68** | **0.002** | **131.39 ± 11.26** | **133.11 ± 13.06** | **0.074** |
| **Asleep** | **114.38 ± 12.74** | **112.74 ± 13.42** | **0.009** | **114.46 ± 10.21** | **112.56 ± 11.33** | **0.003** | **120.14 ± 14.46** | **118.25 ± 16.64** | **0.091** |
| **Diastolic BP (mmHg)** | | | |  |  |  |  |  |  |
| **24-h** | **78.76 ± 6.61** | **80.43 ± 7.19** | **0.007** | **74.13 ± 4.71** | **75.60 ± 5.49** | **0.004** | **72.38 ± 5.89** | **73.26 ± 6.83** | **0.051** |
| **Awake** | **82.02 ± 6.82** | **84.48 ± 7.95** | **0.003** | **76.74 ± 4.98** | **79.15 ± 5.87** | **<0.001** | **74.56 ± 5.51** | **76.50 ± 6.60** | **0.001** |
| **Asleep** | **71.67 ± 8.83** | **71.10 ± 8.83** | **0.085** | **68.25 ± 5.43** | **67.36 ± 5.68** | **0.014** | **68.04 ± 9.12** | **67.01 ± 10.22** | **0.026** |
| **Nocturnal Dip** | | | |  |  |  |  |  |  |
| **Dipping (%)** | **11.0 ± 7.9** | **13.5 ± 9.2** | **0.003** | **9.6 ± 6.6** | **13.4 ± 7.5** | **<0.001** | **8.3 ± 9.8** | **10.9 ± 10.8** | **<0.001** |

**BP, blood pressure; ABPM, ambulatory blood pressure monitoring**

**Supplementary table 6 summary of the agreement statistics of awake-asleep BP change between Alysis and conventional cuff ABPM stratified by age tertile**

**Age T1 (20-69 y.o.)**

| **Metric** | **N** | **Pearson_r** | **Mean_Diff** | **SD_Diff** | **ICC** | **ICC_Lower** | **ICC_Upper** |
| --- | --- | --- | --- | --- | --- | --- | --- |
| **Awake-Sleep SBP change** | **26** | **0.903** | **3.6** | **5.54** | **0.848** | **0.58** | **0.939** |
| **Awake-Sleep DBP change** | **26** | **0.89** | **3.04** | **3.95** | **0.823** | **0.445** | **0.932** |

**Age T2 (70-77 y.o.)**

| **Metric** | **N** | **Pearson_r** | **Mean_Diff** | **SD_Diff** | **ICC** | **ICC_Lower** | **ICC_Upper** |
| --- | --- | --- | --- | --- | --- | --- | --- |
| **Awake-Sleep SBP change** | **26** | **0.845** | **5.32** | **5.5** | **0.724** | **0.138** | **0.899** |
| **Awake-Sleep DBP change** | **26** | **0.747** | **3.29** | **3.91** | **0.631** | **0.127** | **0.845** |

**Age T3 (78-89 y.o.)**

| **Metric** | **N** | **Pearson_r** | **Mean_Diff** | **SD_Diff** | **ICC** | **ICC_Lower** | **ICC_Upper** |
| --- | --- | --- | --- | --- | --- | --- | --- |
| **Awake-Sleep SBP change** | **26** | **0.958** | **3.6** | **4.88** | **0.922** | **0.713** | **0.972** |
| **Awake-Sleep DBP change** | **26** | **0.942** | **2.97** | **2.98** | **0.878** | **0.38** | **0.961** |

**BP, blood pressure; ABPM, ambulatory blood pressure monitoring; SBP, systolic blood pressure; DBP, diastolic blood pressure; Diff, difference; SD, standard deviation; ICC, Intraclass Correlation Coefficient**

**Supplementary table 7 mean 24-h, daytime, and night-time BP as obtained by Alysis and conventional ABPM stratified by BMI tertile (N=78)**

|  | **BMI T1 (17.5-22.5 Kg/m²)**  **N=26** | | | **BMI T2 (22.7-25.5 Kg/m²)**  **N=26** | | | **BMI T3 (25.5-32.1 Kg/m²)**  **N=26** | | |
| --- | --- | --- | --- | --- | --- | --- | --- | --- | --- |
|  | **Alysis** | **ABPM** | **P value** | **Alysis** | **ABPM** | **P value** | **Alysis** | **ABPM** | **P value** |
| **Systolic BP (mmHg)** | | | |  |  |  |  |  |  |
| **24-h** | **123.00 ± 8.51** | **125.41 ± 10.22** | **0.011** | **123.15 ± 11.16** | **123.31 ± 12.44** | **0.868** | **128.59 ± 7.95** | **129.42 ± 8.79** | **0.019** |
| **Awake** | **127.28 ± 10.04** | **130.88 ± 11.02** | **0.003** | **127.81 ± 11.14** | **129.71 ± 13.14** | **0.082** | **131.65 ± 8.36** | **133.25 ± 9.71** | **0.004** |
| **Asleep** | **114.19 ± 9.64** | **113.16 ± 11.37** | **0.06** | **113.01 ± 14.88** | **109.56 ± 16.35** | **0.004** | **121.78 ± 11.69** | **120.83 ± 11.90** | **0.035** |
| **Diastolic BP (mmHg)** | | | |  |  |  |  |  |  |
| **24-h** | **73.31 ± 5.80** | **75.38 ± 7.55** | **0.003** | **73.65 ± 5.86** | **74.87 ± 6.70** | **0.018** | **78.31 ± 6.24** | **79.04 ± 6.60** | **0.009** |
| **Awake** | **75.85 ± 6.23** | **79.00 ± 7.94** | **<0.001** | **76.82 ± 6.02** | **79.24 ± 7.42** | **<0.001** | **80.64 ± 6.62** | **81.89 ± 7.22** | **0.004** |
| **Asleep** | **68.08 ± 6.96** | **67.32 ± 7.80** | **0.017** | **66.73 ± 8.27** | **65.54 ± 8.79** | **0.023** | **73.16 ± 7.65** | **72.61 ± 7.68** | **0.054** |
| **NocturnalDip** | | | |  |  |  |  |  |  |
| **Dipping (%)** | **10.0 ± 7.4** | **13.3 ± 7.4** | **<0.001** | **11.5 ± 8.5** | **15.3 ± 10.5** | **<0.001** | **7.4 ± 8.4** | **9.1 ± 8.8** | **0.003** |

**BP, blood pressure; ABPM, ambulatory blood pressure monitoring; BMI, body mass index**

**Supplementary table 8 summary of the agreement statistics of awake-asleep BP change between Alysis and conventional cuff ABPM stratified by BMI tertile.**

**BMI T1 (17.5-22.5 Kg/m²)**

| **Metric** | **N** | **Pearson_r** | **Mean_Diff** | **SD_Diff** | **ICC** | **ICC_Lower** | **ICC_Upper** |
| --- | --- | --- | --- | --- | --- | --- | --- |
| **Awake-Sleep SBP change** | **26** | **0.876** | **4.62** | **5.11** | **0.798** | **0.288** | **0.928** |
| **Awake-Sleep DBP change** | **26** | **0.836** | **3.9** | **3.34** | **0.686** | **0.001** | **0.892** |

**BMI T2 (22.7-25.5 Kg/m²)**

| **Metric** | **N** | **Pearson_r** | **Mean_Diff** | **SD_Diff** | **ICC** | **ICC_Lower** | **ICC_Upper** |
| --- | --- | --- | --- | --- | --- | --- | --- |
| **Awake-Sleep SBP change** | **26** | **0.926** | **5.36** | **6.45** | **0.835** | **0.42** | **0.94** |
| **Awake-Sleep DBP change** | **26** | **0.896** | **3.6** | **4.24** | **0.806** | **0.347** | **0.929** |

**BMI T3 (25.5-32.1 Kg/m²)**

| **Metric** | **N** | **Pearson_r** | **Mean_Diff** | **SD_Diff** | **ICC** | **ICC_Lower** | **ICC_Upper** |
| --- | --- | --- | --- | --- | --- | --- | --- |
| **Awake-Sleep SBP change** | **26** | **0.949** | **2.55** | **3.82** | **0.924** | **0.755** | **0.971** |
| **Awake-Sleep DBP change** | **26** | **0.931** | **1.79** | **2.84** | **0.902** | **0.714** | **0.961** |

**BP, blood pressure; ABPM, ambulatory blood pressure monitoring; SBP, systolic blood pressure; DBP, diastolic blood pressure; Diff, difference; SD, standard deviation; ICC, Intraclass Correlation Coefficient ; BMI, body mass index**

**Supplementary table 9. mean 24-h, daytime, and night-time BP as obtained by Alysis and conventional ABPM among those who taking CCB (N=61).**

|  | **Alysis** | **ABPM** | **Difference** | **P value** |
| --- | --- | --- | --- | --- |
| **Systolic BP (mmHg)** | | | | |
| **24-h** | **125.28 ± 9.84** | **126.47 ± 10.68** | **-1.19 [-1.95, -0.42]** | **0.003** |
| **Awake** | **129.28 ± 10.15** | **131.59 ± 11.24** | **-2.31 [-3.34, -1.27]** | **<0.001** |
| **Asleep** | **116.77 ± 13.46** | **115.26 ± 14.73** | **1.51 [0.73, 2.28]** | **<0.001** |
| **Diastolic BP (mmHg)** | | | | |
| **24-h** | **75.08 ± 6.23** | **76.18 ± 6.70** | **-1.10 [-1.61, -0.59]** | **<0.001** |
| **Awake** | **77.71 ± 6.32** | **79.59 ± 6.89** | **-1.88 [-2.55, -1.21]** | **<0.001** |
| **Asleep** | **69.48 ± 8.39** | **68.71 ± 8.98** | **0.77 [0.31, 1.22]** | **0.001** |
| **Nocturnal Dip** | | | | |
| **Dipping (%)** | **9.6 ± 8.5** | **12.2 ± 9.6** | **-2.7 [-3.5, -1.8]** | **<0.001** |

**BP, blood pressure; ABPM, ambulatory blood pressure monitoring; CCB, calcium channel blocker**

**Supplementary table 10 summary of the agreement statistics of awake-asleep BP change between Alysis and conventional cuff ABPM among those who are taking CCB.**

| **Metric** | **N** | **Pearson_r** | **Mean_Diff** | **SD_Diff** | **ICC** | **ICC_Lower** | **ICC_Upper** |
| --- | --- | --- | --- | --- | --- | --- | --- |
| **Awake-Sleep SBP change** | **61** | **0.931** | **3.81** | **5.06** | **0.878** | **0.616** | **0.948** |
| **Awake-Sleep DBP change** | **61** | **0.92** | **2.64** | **3.15** | **0.858** | **0.501** | **0.942** |

**BP, blood pressure; ABPM, ambulatory blood pressure monitoring; SBP, systolic blood pressure; DBP, diastolic blood pressure; Diff, difference; SD, standard deviation; ICC, Intraclass Correlation Coefficient ; CCB, calcium channel blocker**

**Supplementary table 11 mean 24-h, daytime, and night-time BP as obtained Alysis and conventional ABPM among those who are taking ARB/ACEi (N=36).**

|  | **Alysis** | **ABPM** | **Difference** | **P value** |
| --- | --- | --- | --- | --- |
| **Systolic BP (mmHg)** | | | | |
| **24-h** | **123.67 ± 10.09** | **124.35 ± 11.94** | **-0.68 [-2.17, 0.80]** | **0.357** |
| **Awake** | **127.86 ± 10.16** | **130.06 ± 12.36** | **-2.20 [-3.82, -0.59]** | **0.009** |
| **Asleep** | **114.50 ± 13.53** | **111.91 ± 15.32** | **2.59 [0.96, 4.21]** | **0.003** |
| **Diastolic BP (mmHg)** | | | | |
| **24-h** | **74.27 ± 5.57** | **75.28 ± 5.91** | **-1.01 [-1.66, -0.36]** | **0.003** |
| **Awake** | **77.03 ± 5.43** | **79.01 ± 5.85** | **-1.98 [-2.91, -1.05]** | **<0.001** |
| **Asleep** | **68.32 ± 8.26** | **67.30 ± 8.75** | **1.02 [0.37, 1.67]** | **0.003** |
| **Nocturnal Dip** | | | | |
| **Dipping (%)** | **10.4 ± 8.2** | **13.8 ± 9.4** | **-3.4 [-4.7, -2.1]** | **<0.001** |

**BP, blood pressure; ABPM, ambulatory blood pressure monitoring ; ARB, angiotensin receptor blocker；ACEi, angiotensin converting enzyme inhibitor**

**Supplementary table 12 summary of the agreement statistics of awake-asleep BP change between Alysis and conventional cuff ABPM among those who are taking ARB/ACEi.**

| **Metric** | **N** | **Pearson_r** | **Mean_Diff** | **SD_Diff** | **ICC** | **ICC_Lower** | **ICC_Upper** |
| --- | --- | --- | --- | --- | --- | --- | --- |
| **Awake-Sleep SBP change** | **36** | **0.921** | **4.79** | **5.5** | **0.844** | **0.423** | **0.942** |
| **Awake-Sleep DBP change** | **36** | **0.87** | **3** | **3.75** | **0.799** | **0.395** | **0.918** |

**BP, blood pressure; ABPM, ambulatory blood pressure monitoring; SBP, systolic blood pressure; DBP, diastolic blood pressure; Diff, difference; SD, standard deviation; ICC, Intraclass Correlation Coefficient; ARB, angiotensin receptor blocker；ACEi, angiotensin converting enzyme inhibitor**

**Supplementary table 13 mean 24-h, daytime, and night-time BP as obtained by Alysis and**

**conventional ABPM among those who are taking diuretics (N=6).**

|  | **Alysis** | **ABPM** | **Difference** | **P value** |
| --- | --- | --- | --- | --- |
| **Systolic BP (mmHg)** | | | | |
| **24-h** | **123.10 ± 6.04** | **121.30 ± 11.59** | **1.80 [-6.49, 10.09]** | **0.601** |
| **Awake** | **129.79 ± 8.50** | **129.73 ± 12.56** | **0.05 [-5.79, 5.90]** | **0.982** |
| **Asleep** | **108.64 ± 6.27** | **103.54 ± 8.79** | **5.10 [-5.86, 16.06]** | **0.285** |
| **Diastolic BP (mmHg)** | | | | |
| **24-h** | **75.06 ± 3.45** | **74.58 ± 4.83** | **0.49 [-1.35, 2.32]** | **0.526** |
| **Awake** | **79.43 ± 4.53** | **79.98 ± 5.83** | **-0.54 [-4.36, 3.27]** | **0.729** |
| **Asleep** | **65.64 ± 2.60** | **63.51 ± 2.83** | **2.13 [-0.58, 4.85]** | **0.1** |
| **Nocturnal Dip** | | | | |
| **Dipping (%)** | **16.0 ± 7.5** | **19.9 ± 6.4** | **-3.9 [-9.5, 1.7]** | **0.132** |

**BP, blood pressure; ABPM, ambulatory blood pressure monitoring**

**Supplementary table 14. summary of the agreement statistics of awake-asleep BP change between Alysis and conventional ABPM among those who are taking diuretics.**

| **Metric** | **N** | **Pearson_r** | **Mean_Diff** | **SD_Diff** | **ICC** | **ICC_Lower** | **ICC_Upper** |
| --- | --- | --- | --- | --- | --- | --- | --- |
| **Awake-Sleep SBP change** | **6** | **0.833** | **5.05** | **6.05** | **0.765** | **0.071** | **0.963** |
| **Awake-Sleep DBP change** | **6** | **0.578** | **2.68** | **5.88** | **0.566** | **-0.224** | **0.923** |

**BP, blood pressure; ABPM, ambulatory blood pressure monitoring; SBP, systolic blood pressure; DBP, diastolic blood pressure; Diff, difference; SD, standard deviation; ICC, Intraclass Correlation Coefficient**

**Supplementary table 15 mean 24-h, daytime, and night-time BP as obtained by Alysis and conventional ABPM among those who are taking beta blocker (N=12).**

|  | **Alysis** | **ABPM** | **Difference** | **P value** |
| --- | --- | --- | --- | --- |
| **Systolic BP (mmHg)** | | | | |
| **24-h** | **120.50 ± 7.52** | **119.58 ± 9.62** | **0.92 [-2.67, 4.50]** | **0.585** |
| **Awake** | **124.11 ± 6.46** | **124.63 ± 8.19** | **-0.52 [-3.58, 2.55]** | **0.717** |
| **Asleep** | **113.65 ± 10.54** | **110.67 ± 13.40** | **2.98 [-1.91, 7.87]** | **0.207** |
| **Diastolic BP (mmHg)** | | | | |
| **24-h** | **73.26 ± 5.99** | **74.16 ± 6.88** | **-0.90 [-2.31, 0.52]** | **0.191** |
| **Awake** | **75.93 ± 5.20** | **78.10 ± 6.16** | **-2.17 [-3.94, -0.39]** | **0.021** |
| **Asleep** | **68.28 ± 8.39** | **67.31 ± 9.74** | **0.97 [-0.97, 2.91]** | **0.296** |
| **Nocturnal Dip** | | | | |
| **Dipping (%)** | **8.5 ± 4.8** | **11.3 ± 7.3** | **-2.8 [-5.8, 0.3]** | **0.069** |

**BP, blood pressure; ABPM, ambulatory blood pressure monitoring**

**Supplementary table 16 summary of the agreement statistics of awake-asleep BP change between Alysis and conventional ABPM among those who taking beta-blocker.**

| **Metric** | **N** | **Pearson_r** | **Mean_Diff** | **SD_Diff** | **ICC** | **ICC_Lower** | **ICC_Upper** |
| --- | --- | --- | --- | --- | --- | --- | --- |
| **Awake-Sleep SBP change** | **12** | **0.792** | **3.5** | **5.83** | **0.669** | **0.189** | **0.892** |
| **Awake-Sleep DBP change** | **12** | **0.793** | **3.13** | **3.72** | **0.663** | **0.08** | **0.896** |

**BP, blood pressure; ABPM, ambulatory blood pressure monitoring; SBP, systolic blood pressure; DBP, diastolic blood pressure; Diff, difference; SD, standard deviation; ICC, Intraclass Correlation Coefficient**

**Supplementary table 17 mean 24-h, daytime, and night-time BP as obtained by Alysis and**

**conventional ABPM among those who are taking mineralocorticoid receptor antagonist (N=19).**

|  | **Alysis** | **ABPM** | **Difference** | **P value** |
| --- | --- | --- | --- | --- |
| **Systolic BP (mmHg)** | | | | |
| **24-h** | **126.04 ± 8.43** | **126.69 ± 8.07** | **-0.65 [-2.02, 0.73]** | **0.336** |
| **Awake** | **130.48 ± 9.09** | **131.83 ± 8.49** | **-1.35 [-3.39, 0.69]** | **0.181** |
| **Asleep** | **117.08 ± 11.22** | **115.70 ± 10.77** | **1.38 [0.26, 2.50]** | **0.018** |
| **Diastolic BP (mmHg)** | | | | |
| **24-h** | **76.52 ± 4.70** | **77.94 ± 5.91** | **-1.42 [-2.90, 0.06]** | **0.059** |
| **Awake** | **79.94 ± 5.30** | **82.24 ± 6.49** | **-2.30 [-4.13, -0.46]** | **0.017** |
| **Asleep** | **69.51 ± 5.14** | **68.64 ± 5.54** | **0.88 [-0.05, 1.80]** | **0.061** |
| **Nocturnal Dip** | | | | |
| **Dipping (%)** | **10.1 ± 7.6** | **12.1 ± 7.5** | **-2.0 [-3.6, -0.4]** | **0.019** |

**BP, blood pressure; ABPM, ambulatory blood pressure monitoring**

**Supplementary table 18. summary of the agreement statistics of awake-asleep BP change between Alysis and conventional ABPM among those who taking mineralocorticoid receptor antagonist.**

| **Metric** | **N** | **Pearson_r** | **Mean_Diff** | **SD_Diff** | **ICC** | **ICC_Lower** | **ICC_Upper** |
| --- | --- | --- | --- | --- | --- | --- | --- |
| **Awake-Sleep SBP change** | **19** | **0.896** | **2.73** | **4.69** | **0.87** | **0.636** | **0.951** |
| **Awake-Sleep DBP change** | **19** | **0.755** | **3.18** | **3.54** | **0.633** | **0.074** | **0.862** |

**BP, blood pressure; ABPM, ambulatory blood pressure monitoring; SBP, systolic blood pressure; DBP, diastolic blood pressure; Diff, difference; SD, standard deviation; ICC, Intraclass Correlation Coefficient**

**Supplemental table 19 mean 24-h, daytime, and night-time BP as obtained by Alysis and conventional ABPM among those who are taking angiotensin receptor neprilysin inhibitor (N=15).**

|  | **Alysis** | **ABPM** | **Difference** | **P value** |
| --- | --- | --- | --- | --- |
| **Systolic BP (mmHg)** | | | | |
| **24-h** | **128.81 ± 9.21** | **131.74 ± 9.70** | **-2.94 [-5.38, -0.49]** | **0.022** |
| **Awake** | **133.57 ± 8.46** | **137.91 ± 9.36** | **-4.34 [-7.50, -1.18]** | **0.011** |
| **Asleep** | **119.37 ± 13.79** | **118.50 ± 13.97** | **0.87 [-0.72, 2.46]** | **0.262** |
| **Diastolic BP (mmHg)** | | | | |
| **24-h** | **76.82 ± 7.76** | **78.90 ± 8.83** | **-2.08 [-3.80, -0.36]** | **0.021** |
| **Awake** | **80.11 ± 8.07** | **83.21 ± 9.76** | **-3.10 [-5.40, -0.80]** | **0.012** |
| **Asleep** | **70.02 ± 8.28** | **69.41 ± 8.05** | **0.60 [-0.33, 1.54]** | **0.186** |
| **Nocturnal Dip** | | | | |
| **Dipping (%)** | **10.7 ± 8.0** | **14.0 ± 8.9** | **-3.3 [-5.7, -1.0]** | **0.009** |

**BP, blood pressure; ABPM, ambulatory blood pressure monitoring**

**Supplemental table 20 summary of the agreement statistics of awake-asleep BP change between Alysis and conventional cuff ABPM among those who are taking angiotensin receptor neprilysin inhibitor.**

| **Metric** | **N** | **Pearson_r** | **Mean_Diff** | **SD_Diff** | **ICC** | **ICC_Lower** | **ICC_Upper** |
| --- | --- | --- | --- | --- | --- | --- | --- |
| **Awake-Sleep SBP change** | **15** | **0.834** | **5.21** | **6.83** | **0.754** | **0.274** | **0.919** |
| **Awake-Sleep DBP change** | **15** | **0.846** | **3.71** | **4.5** | **0.745** | **0.219** | **0.917** |

**BP, blood pressure; ABPM, ambulatory blood pressure monitoring; SBP, systolic blood pressure; DBP, diastolic blood pressure; Diff, difference; SD, standard deviation; ICC, Intraclass Correlation Coefficient**

**Supplemental table 21 mean 24-h, daytime, and night-time BP as obtained by Alysis and conventional ABPM among those with daytime hypertension (N=37).**

|  | **Alysis** | **ABPM** | **Difference** | **P value** |
| --- | --- | --- | --- | --- |
| **Systolic BP (mmHg)** | | | | |
| **24-h** | **131.13 ± 8.54** | **133.50 ± 8.89** | **-2.36 [-3.77, -0.95]** | **0.002** |
| **Awake** | **136.10 ± 8.28** | **139.88 ± 8.86** | **-3.78 [-5.59, -1.96]** | **<0.001** |
| **Asleep** | **120.72 ± 13.65** | **119.33 ± 14.87** | **1.40 [0.43, 2.36]** | **0.006** |
| **Diastolic BP (mmHg)** | | | | |
| **24-h** | **78.02 ± 5.90** | **79.90 ± 7.03** | **-1.88 [-2.93, -0.84]** | **<0.001** |
| **Awake** | **81.23 ± 5.96** | **84.08 ± 7.28** | **-2.85 [-4.14, -1.56]** | **<0.001** |
| **Asleep** | **71.30 ± 8.30** | **70.59 ± 8.99** | **0.70 [0.09, 1.32]** | **0.026** |
| **Nocturnal Dip** | | | | |
| **Dipping (%)** | **11.3 ± 8.6** | **14.6 ± 9.7** | **-3.3 [-4.5, -2.2]** | **<0.001** |

**BP, blood pressure; ABPM, ambulatory blood pressure monitoring**

**Supplemental table 22 summary of the agreement statistics of awake-asleep BP change between Alysis and conventional ABPM among those with daytime hypertension.**

| **Metric** | **N** | **Pearson_r** | **Mean_Diff** | **SD_Diff** | **ICC** | **ICC_Lower** | **ICC_Upper** |
| --- | --- | --- | --- | --- | --- | --- | --- |
| **Awake-Sleep SBP change** | **37** | **0.928** | **5.17** | **5.57** | **0.852** | **0.391** | **0.947** |
| **Awake-Sleep DBP change** | **37** | **0.904** | **3.55** | **3.58** | **0.816** | **0.265** | **0.935** |

**BP, blood pressure; ABPM, ambulatory blood pressure monitoring; SBP, systolic blood pressure; DBP, diastolic blood pressure; Diff, difference; SD, standard deviation; ICC, Intraclass Correlation Coefficient**

**Supplementary table 23 mean 24-h, daytime, and night-time BP as obtained by Alysis and conventional ABPM among those with nighttime hypertension (N=37).**

|  | **Alysis** | **ABPM** | **Difference** | **P value** |
| --- | --- | --- | --- | --- |
| **Systolic BP (mmHg)** | | | | |
| **24-h** | **130.24 ± 9.02** | **131.78 ± 9.95** | **-1.54 [-2.88, -0.20]** | **0.026** |
| **Awake** | **132.28 ± 9.81** | **134.69 ± 10.81** | **-2.40 [-4.11, -0.70]** | **0.007** |
| **Asleep** | **126.51 ± 10.02** | **125.74 ± 10.85** | **0.76 [-0.11, 1.64]** | **0.084** |
| **Diastolic BP (mmHg)** | | | | |
| **24-h** | **78.17 ± 5.43** | **79.76 ± 6.31** | **-1.59 [-2.56, -0.61]** | **0.002** |
| **Awake** | **79.63 ± 5.84** | **82.01 ± 6.89** | **-2.38 [-3.59, -1.16]** | **<0.001** |
| **Asleep** | **75.47 ± 6.05** | **75.07 ± 6.80** | **0.40 [-0.22, 1.02]** | **0.203** |
| **Nocturnal Dip** | | | | |
| **Dipping (%)** | **4.2 ± 6.1** | **6.4 ± 7.0** | **-2.2 [-3.3, -1.2]** | **<0.001** |

**BP, blood pressure; ABPM, ambulatory blood pressure monitoring**

**Supplementary table 24 summary of the agreement statistics of awake-asleep BP change between Alysis and conventional cuff ABPM among those with nighttime hypertension.**

| **Metric** | **N** | **Pearson_r** | **Mean_Diff** | **SD_Diff** | **ICC** | **ICC_Lower** | **ICC_Upper** |
| --- | --- | --- | --- | --- | --- | --- | --- |
| **Awake-Sleep SBP change** | **37** | **0.871** | **3.17** | **4.84** | **0.813** | **0.532** | **0.916** |
| **Awake-Sleep DBP change** | **37** | **0.841** | **2.78** | **3.54** | **0.742** | **0.318** | **0.889** |

**BP, blood pressure; ABPM, ambulatory blood pressure monitoring; SBP, systolic blood pressure; DBP, diastolic blood pressure; Diff, difference; SD, standard deviation; ICC, Intraclass Correlation Coefficient**

**Supplementary table 25 mean 24-h, daytime, and night-time BP as obtained by Alysis and conventional ABPM among those with 24-h SBP≧130mmHg (N=30).**

|  | **Alysis** | **ABPM** | **Difference** | **P value** |
| --- | --- | --- | --- | --- |
| **Systolic BP (mmHg)** | | | | |
| **24-h** | **134.48 ± 6.43** | **136.92 ± 6.59** | **-2.44 [-3.96, -0.92]** | **0.003** |
| **Awake** | **138.58 ± 6.73** | **142.27 ± 7.46** | **-3.69 [-5.66, -1.72]** | **<0.001** |
| **Asleep** | **125.85 ± 12.78** | **124.96 ± 13.70** | **0.89 [-0.16, 1.93]** | **0.094** |
| **Diastolic BP (mmHg)** | | | | |
| **24-h** | **77.78 ± 6.12** | **79.32 ± 7.33** | **-1.54 [-2.61, -0.47]** | **0.006** |
| **Awake** | **80.48 ± 6.16** | **82.89 ± 7.77** | **-2.41 [-3.73, -1.09]** | **<0.001** |
| **Asleep** | **72.07 ± 8.70** | **71.35 ± 9.44** | **0.73 [0.02, 1.43]** | **0.043** |
| **Nocturnal Dip** | | | | |
| **Dipping rate** | **9.0 ± 9.4** | **11.9 ± 10.5** | **-2.9 [-4.0, -1.7]** | **<0.001** |

**BP, blood pressure; ABPM, ambulatory blood pressure monitoring; SBP, systolic blood pressure**

**Supplementary table 26 summary of the agreement statistics of awake-asleep BP change between Alysis and conventional cuff among those with 24-h SBP≧130mmHg**

| **Metric** | **N** | **Pearson_r** | **Mean_Diff** | **SD_Diff** | **ICC** | **ICC_Lower** | **ICC_Upper** |
| --- | --- | --- | --- | --- | --- | --- | --- |
| **Awake-Sleep SBP change** | **30** | **0.95** | **4.58** | **5.31** | **0.895** | **0.552** | **0.963** |
| **Awake-Sleep DBP change** | **30** | **0.942** | **3.14** | **3.29** | **0.869** | **0.402** | **0.956** |

**BP, blood pressure; SBP, systolic blood pressure; DBP, diastolic blood pressure; Diff, difference; SD, standard deviation; ICC, Intraclass Correlation Coefficient**

**Supplementary table 27 mean 24-h, daytime, and night-time BP as obtained by Alysis and conventional ABPM among those with 24-h SBP120-130mmHg (N=21)**

|  | **Alysis** | **ABPM** | **Difference** | **P value** |
| --- | --- | --- | --- | --- |
| **Systolic BP (mmHg)** | | | | |
| **24-h** | **123.47 ± 3.39** | **125.36 ± 2.06** | **-1.89 [-3.32, -0.47]** | **0.012** |
| **Awake** | **127.26 ± 4.79** | **130.21 ± 4.84** | **-2.95 [-4.96, -0.94]** | **0.006** |
| **Asleep** | **115.36 ± 8.12** | **114.34 ± 9.48** | **1.02 [-0.19, 2.23]** | **0.095** |
| **Diastolic BP (mmHg)** | | | | |
| **24-h** | **76.00 ± 5.39** | **77.50 ± 6.17** | **-1.49 [-2.85, -0.13]** | **0.033** |
| **Awake** | **78.51 ± 5.85** | **80.82 ± 6.70** | **-2.31 [-4.07, -0.55]** | **0.013** |
| **Asleep** | **70.64 ± 7.43** | **69.90 ± 8.12** | **0.74 [0.04, 1.44]** | **0.039** |
| **Nocturnal Dip** | | | | |
| **Dipping (%)** | **9.1 ± 8.7** | **11.8 ± 10.3** | **-2.7 [-4.6, -0.9]** | **0.006** |

**BP, blood pressure; ABPM, ambulatory blood pressure monitoring**

**Supplementary table 28 summary of the agreement statistics of awake-asleep BP change between Alysis and conventional cuff among those with 24-h SBP 120-130mmHg**

| **Metric** | **N** | **Pearson_r** | **Mean_Diff** | **SD_Diff** | **ICC** | **ICC_Lower** | **ICC_Upper** |
| --- | --- | --- | --- | --- | --- | --- | --- |
| **Awake-Sleep SBP change** | **21** | **0.91** | **3.96** | **5.89** | **0.853** | **0.559** | **0.945** |
| **Awake-Sleep DBP change** | **21** | **0.872** | **3.05** | **4.03** | **0.811** | **0.415** | **0.932** |

**BP, blood pressure; SBP, systolic blood pressure; DBP, diastolic blood pressure; Diff, difference; SD, standard deviation; ICC, Intraclass Correlation Coefficient**

**Supplementary table 29 mean 24-h, daytime, and night-time BP as obtained by Alysis and conventional ABPM among those with 24-h SBP<120mmHg (N=27)**

|  | **Alysis** | **ABPM** | **Difference** | **P value** |
| --- | --- | --- | --- | --- |
| **Systolic BP (mmHg)** | | | | |
| **24-h** | **115.41 ± 3.83** | **114.51 ± 4.02** | **0.90 [-0.64, 2.45]** | **0.241** |
| **Awake** | **119.47 ± 4.91** | **119.91 ± 5.03** | **-0.44 [-1.93, 1.04]** | **0.546** |
| **Asleep** | **106.50 ± 6.35** | **103.05 ± 6.54** | **3.45 [1.39, 5.52]** | **0.002** |
| **Diastolic BP (mmHg)** | | | | |
| **24-h** | **71.39 ± 5.57** | **72.39 ± 5.79** | **-1.00 [-1.60, -0.40]** | **0.002** |
| **Awake** | **74.20 ± 6.03** | **76.27 ± 6.51** | **-2.08 [-3.03, -1.13]** | **<0.001** |
| **Asleep** | **65.24 ± 6.12** | **64.22 ± 6.05** | **1.01 [0.18, 1.84]** | **0.019** |
| **Nocturnal Dip** | | | | |
| **Dipping (%)** | **10.7 ± 6.3** | **13.9 ± 6.8** | **-3.2 [-4.8, -1.6]** | **<0.001** |

**BP, blood pressure; ABPM, ambulatory blood pressure monitoring**

**Supplementary table 30 summary of the agreement statistics of awake-asleep BP change between Alysis and conventional ABPM among those with 24-h SBP<120mmHg**

| **Metric** | **N** | **Pearson_r** | **Mean_Diff** | **SD_Diff** | **ICC** | **ICC_Lower** | **ICC_Upper** |
| --- | --- | --- | --- | --- | --- | --- | --- |
| **Awake-Sleep SBP change** | **27** | **0.821** | **3.89** | **5** | **0.741** | **0.3** | **0.895** |
| **Awake-Sleep DBP change** | **27** | **0.789** | **3.09** | **3.71** | **0.69** | **0.195** | **0.874** |

**BP, blood pressure; SBP, systolic blood pressure; DBP, diastolic blood pressure; Diff, difference; SD, standard deviation; ICC, Intraclass Correlation Coefficient**

**Supplementary table 31 mean 24-h, daytime, and night-time BP as obtained by Alysis and conventional ABPM among those with 24-h DBP≧80mmHg (N=22)**

|  | **Alysis** | **ABPM** | **Difference** | **P value** |
| --- | --- | --- | --- | --- |
| **Systolic BP (mmHg)** | | | | |
| **24-h** | **129.65 ± 8.63** | **131.98 ± 9.92** | **-2.33 [-4.50, -0.17]** | **0.036** |
| **Awake** | **133.47 ± 8.62** | **136.91 ± 10.14** | **-3.43 [-6.23, -0.64]** | **0.018** |
| **Asleep** | **121.57 ± 13.21** | **120.16 ± 14.96** | **1.41 [0.06, 2.76]** | **0.042** |
| **Diastolic BP (mmHg)** | | | | |
| **24-h** | **82.16 ± 3.82** | **85.20 ± 3.40** | **-3.04 [-4.50, -1.59]** | **<0.001** |
| **Awake** | **84.87 ± 4.43** | **88.99 ± 4.21** | **-4.12 [-5.95, -2.29]** | **<0.001** |
| **Asleep** | **76.42 ± 7.16** | **76.22 ± 7.96** | **0.20 [-0.70, 1.10]** | **0.651** |
| **Nocturnal Dip** | | | | |
| **Dipping (%)** | **8.9 ± 8.7** | **12.1 ± 10.3** | **-3.2 [-5.0, -1.4]** | **0.001** |

**BP, blood pressure; ABPM, ambulatory blood pressure monitoring**

**Supplementary table 32 summary of the agreement statistics of awake-asleep BP change between Alysis and conventional cuff among those with 24-h DBP≧80mmHg**

| **Metric** | **N** | **Pearson_r** | **Mean_Diff** | **SD_Diff** | **ICC** | **ICC_Lower** | **ICC_Upper** |
| --- | --- | --- | --- | --- | --- | --- | --- |
| **Awake-Sleep SBP change** | **22** | **0.9** | **4.85** | **6.33** | **0.829** | **0.447** | **0.938** |
| **Awake-Sleep DBP change** | **22** | **0.907** | **4.32** | **4.11** | **0.811** | **0.187** | **0.941** |

**BP, blood pressure; SBP, systolic blood pressure; DBP, diastolic blood pressure; Diff, difference; SD, standard deviation; ICC, Intraclass Correlation Coefficient**

**Supplementary table 33 mean 24-h, daytime, and night-time BP as obtained by Alysis and conventional ABPM among those with 24-h DBP70-80mmHg (N=40)**

|  | **Alysis** | **ABPM** | **Difference** | **P value** |
| --- | --- | --- | --- | --- |
| **Systolic BP (mmHg)** | | | | |
| **24-h** | **124.61 ± 8.99** | **125.00 ± 10.30** | **-0.39 [-1.68, 0.89]** | **0.539** |
| **Awake** | **128.33 ± 9.50** | **130.11 ± 11.05** | **-1.78 [-3.10, -0.45]** | **0.01** |
| **Asleep** | **116.29 ± 12.49** | **113.77 ± 14.01** | **2.52 [1.00, 4.04]** | **0.002** |
| **Diastolic BP (mmHg)** | | | | |
| **24-h** | **74.77 ± 2.43** | **75.54 ± 2.59** | **-0.76 [-1.35, -0.17]** | **0.013** |
| **Awake** | **77.27 ± 2.98** | **78.99 ± 3.45** | **-1.72 [-2.58, -0.87]** | **<0.001** |
| **Asleep** | **69.22 ± 5.58** | **68.02 ± 6.01** | **1.20 [0.60, 1.80]** | **<0.001** |
| **Nocturnal Dip** | | | | |
| **Dipping (%)** | **9.2 ± 8.6** | **12.3 ± 9.8** | **-3.1 [-4.3, -1.9]** | **<0.001** |

**BP, blood pressure; ABPM, ambulatory blood pressure monitoring**

**Supplementary table 34 summary of the agreement statistics of awake-asleep BP change between Alysis and conventional ABPM among those with 24-h DBP 70-80mmHg**

| **Metric** | **N** | **Pearson_r** | **Mean_Diff** | **SD_Diff** | **ICC** | **ICC_Lower** | **ICC_Upper** |
| --- | --- | --- | --- | --- | --- | --- | --- |
| **Awake-Sleep SBP change** | **40** | **0.941** | **4.3** | **4.97** | **0.882** | **0.525** | **0.956** |
| **Awake-Sleep DBP change** | **40** | **0.885** | **2.92** | **3.64** | **0.814** | **0.426** | **0.924** |

**BP, blood pressure; SBP, systolic blood pressure; DBP, diastolic blood pressure; Diff, difference; SD, standard deviation; ICC, Intraclass Correlation Coefficient**

**Supplementary table 35 mean 24-h, daytime, and night-time BP as obtained by Alysis and conventional ABPM among those with 24-h DBP<70mmHg (N=16)**

|  | **Alysis** | **ABPM** | **Difference** | **P value** |
| --- | --- | --- | --- | --- |
| **Systolic BP (mmHg)** | | | | |
| **24-h** | **119.17 ± 9.28** | **120.51 ± 9.65** | **-1.35 [-2.47, -0.23]** | **0.021** |
| **Awake** | **124.10 ± 10.78** | **126.47 ± 11.05** | **-2.36 [-4.22, -0.51]** | **0.016** |
| **Asleep** | **109.21 ± 9.26** | **108.62 ± 10.15** | **0.59 [-0.50, 1.68]** | **0.269** |
| **Diastolic BP (mmHg)** | | | | |
| **24-h** | **66.16 ± 3.31** | **66.61 ± 2.99** | **-0.45 [-1.22, 0.32]** | **0.235** |
| **Awake** | **69.29 ± 4.32** | **70.37 ± 4.13** | **-1.09 [-2.20, 0.03]** | **0.055** |
| **Asleep** | **59.82 ± 3.07** | **59.05 ± 3.07** | **0.77 [0.07, 1.47]** | **0.034** |
| **Nocturnal Dip** | | | | |
| **Dipping rate** | **11.8 ± 6.0** | **13.9 ± 6.4** | **-2.1 [-3.8, -0.4]** | **0.019** |

**BP, blood pressure; ABPM, ambulatory blood pressure monitoring**

**Supplementary table 36 summary of the agreement statistics of awake-asleep BP change between Alysis and conventional ABPM among those with 24-h DBP<70mmHg**

| **Metric** | **N** | **Pearson_r** | **Mean_Diff** | **SD_Diff** | **ICC** | **ICC_Lower** | **ICC_Upper** |
| --- | --- | --- | --- | --- | --- | --- | --- |
| **Awake-Sleep SBP change** | **16** | **0.852** | **2.95** | **4.68** | **0.804** | **0.452** | **0.931** |
| **Awake-Sleep DBP change** | **16** | **0.901** | **1.86** | **2.09** | **0.836** | **0.334** | **0.95** |

**BP, blood pressure; SBP, systolic blood pressure; DBP, diastolic blood pressure; Diff, difference; SD, standard deviation; ICC, Intraclass Correlation Coefficient**
